# A zebrafish-based *in vivo* model of Zika virus infection unveils alterations of the glutamatergic neuronal development and NS4A as a key viral determinant of neuropathogenesis

**DOI:** 10.1371/journal.ppat.1012756

**Published:** 2024-12-02

**Authors:** Aïssatou Aïcha Sow, Priyanka Jamadagni, Pietro Scaturro, Shunmoogum A. Patten, Laurent Chatel-Chaix

**Affiliations:** 1 Centre Armand-Frappier Santé Biotechnologie, Institut National de la Recherche Scientifique, Laval, Québec, Canada; 2 Leibniz Institute of Virology, Hamburg, Germany; 3 Center of Excellence in Research on Orphan Diseases-Fondation Courtois (CERMO-FC), Québec, Canada; 4 Regroupement Intersectoriel de Recherche en Santé de l’Université du Québec (RISUQ), Québec, Canada; 5 Swine and Poultry Infectious Diseases Research Centre (CRIPA), Québec, Canada; University of Minnesota Twin Cities, UNITED STATES OF AMERICA

## Abstract

Infection of pregnant women by Zika virus (ZIKV) is associated with severe neurodevelopmental defects in newborns through poorly defined mechanisms. Here, we established a zebrafish *in vivo* model of ZIKV infection to circumvent limitations of existing mammalian models. Leveraging the unique tractability of this system, we gained unprecedented access to the ZIKV-infected brain at early developmental stages. The infection of zebrafish larvae with ZIKV phenocopied the disease in mammals including a reduced head area and neural progenitor cells (NPC) infection and depletion. Moreover, transcriptomic analyses of NPCs isolated from ZIKV-infected embryos revealed a distinct dysregulation of genes involved in survival and neuronal differentiation, including downregulation of the expression of the glutamate transporter *vglut1*, resulting in an altered glutamatergic network in the brain. Mechanistically, ectopic expression of ZIKV protein NS4A in the larvae recapitulated the morphological defects observed in infected animals, identifying NS4A as a key determinant of neurovirulence and a promising antiviral target for developing therapies.

## Author summary

Infections with Zika virus (ZIKV) constitute a major global public health concern worldwide. Indeed, infection of pregnant women can induce severe defects of the fetal brain and, eventually, microcephaly in the infant. Most importantly, no antiviral therapies or vaccines against ZIKV are currently available, as we poorly understand how the disease develops.

Addressing these questions in mammalian models is highly challenging. Here, we report an alternative *in vivo* model of ZIKV infection based on zebrafish which circumvents limitations of existing models. Leveraging the unique tractability of this system, we gained unprecedented access to the ZIKV-infected brain during early developmental stages at the tissue, cell and molecular levels. The infection of zebrafish larvae with ZIKV phenocopied the disease in mammals including a reduced head area and neural progenitor cells (NPC) infection and depletion. This correlated with a transcriptomic reprogramming of NPCs and alterations of the excitatory glutamatergic neuronal networks. Furthermore, we identified ZIKV NS4A as a key determinant of neuropathogenesis, supporting that this viral protein constitutes a promising antiviral target for developing therapies.

## Introduction

Since its introduction and emergence in Latin America in 2015, Zika virus (ZIKV) constitutes a public health concern, especially considering that neither antiviral treatments nor vaccines against this orthoflavivirus are currently available. ZIKV is mainly transmitted through mosquito bite but unlike most orthoflaviviruses, it can also be vertically transmitted to the fetus in infected pregnant women. ZIKV may reach the fetal brain and cause major neurodevelopment defects leading to severe birth defects and life-long disabilities. This affliction, named congenital Zika syndrome (CZS), includes microcephaly, brain damage and body mobility restriction. Even asymptomatic infected women (80% of the cases) are at risk of delivering newborns with long-term neurological/cognitive problems [1]. Notably, children that were exposed to ZIKV *in utero* but without CZS at birth may nevertheless show neurodevelopmental delays within the first two years of life, particularly in cognitive and language development [2]. These symptoms can be explained by the fact that following congenital transmission, ZIKV infects neural progenitor cells (NPC) in the fetal brain resulting in an alteration of their differentiation program as well as in their apoptosis-driven cell death through poorly defined mechanisms [3–7]. In addition, ZIKV also infects other cell types of the brain such as astrocytes and microglial cells, potentially inducing neuroinflammation and thereby, indirectly contributing to the brain developmental defects [8]. Ultimately, brain infection leads to severe defects in neuronal maturation resulting in cortical thinning, growth restriction and thus, reduced size of the brain. Importantly, the viral and host determinants driving ZIKV neuropathogenesis are largely poorly understood.

Upon ZIKV entry into the target cell, the positive-sense viral RNA (vRNA) genome is translated into a polyprotein which is subsequently cleaved into 10 mature viral proteins. The seven non-structural (NS) proteins are responsible for vRNA replication while structural proteins capsid (C), pre-membrane (prM) and envelope (E), together with the viral genome, orchestrate the assembly of new viral particles [9,10]. Flaviviral NS4A and NS4B are of particular interest since they are absolutely required for replication through poorly defined processes. In the case of ZIKV, they were reported to inhibit the growth of NPC-derived neurospheres *in vitro* alone or in combination [11–16]. These transmembrane proteins lack enzymatic activities and interact together in the endoplasmic reticulum (ER) suggesting that they share functions in viral replication. Reverse genetics and pharmacological approaches recently demonstrated that NS4A of ZIKV and dengue virus (DENV, closely related to ZIKV) contribute to the biogenesis of ER-derived replication organelles which host the vRNA synthesis process [12,17]. In addition, the NS4A-NS4B precursor is also believed to play specific roles in flavivirus life cycle. In the case of DENV, NS4A-2K-NS4B interacts specifically with NS1 viral protein to regulate viral replication [18]. Interestingly, DENV NS4B (most likely as a precursor in some cases) is the target of several highly potent antivirals, including two currently in phase 2 clinical trials [19–24]. Furthermore, individual expression of ZIKV NS4A and NS4B can inhibit the AKT/mTOR pathway and interfere with the early induction of type I interferon [11,25]. Finally, ubiquitous expression of ZIKV NS4A in the invertebrate model of drosophila led to marked decrease in the size of the third instar larval brain [26,27], although such impact remains to be confirmed in a vertebrate model whose CNS development resembles more closely the one of higher mammals.

Murine infection models and organoid culture technology contributed to better understand ZIKV neurotropism and neurovirulence. Infected immunodeficient adult mice or pups show accumulation of ZIKV in both the brain and the spinal cord in addition to other organs such as liver, testes and spleen [3,6,7,28]. However, the individual contribution of ZIKV proteins to neurovirulence was barely addressed *in vivo* in vertebrate models. Furthermore, monitoring the physiology of NPCs in the whole brain of vertebrates, especially in transgenic mammalian models remains challenging because of high costs, invested time, ethical considerations, access to specific cell types inside the brain for imaging and omic analysis, and limited genetic plasticity associated to potential embryonic lethality. Thus, alternative models more conducive to the study of early development of the ZIKV-infected brain are required.

The zebrafish has emerged as a powerful and cost-effective tool for studying neurological diseases relevant to humans [29,30]. The zebrafish embryo (1-to-2 day-old) and larva (3-to-6 day-old) are ideally suited to examine neurodevelopment in the brain as the neural population, connectivity and axon tracts as a whole are preserved during experimentation. The optically transparent fish represents an exquisite *in vivo* toolbox enabling easy imaging of the brain in transgenic animals, loss- or gain-of-function genetic approaches, behavioral tests to examine changes in motor activity, and the ease to simultaneously use hundreds of developing animals on a short time frame (generally a few days). Furthermore, the larval zebrafish brain shares basic neuroanatomical layout with that of mammals [31]. Finally, zebrafish is permissive to several human viruses (*e*.*g*., norovirus, herpes simplex virus, Rift Valley fever virus [24,32,33]) and phenocopies several human neuropathological diseases such as amyotrophic lateral sclerosis, fronto-temporal dementia and CHARGE syndrome [29,30,34–36].

In this study, we have established an innovative zebrafish-based ZIKV infection model to study viral neuropathogenesis *in vivo*. In this system, larvae infected with ZIKV exhibited severe morphological defects during their development. ZIKV infection was detected in brain NPCs, which correlated with a decrease in their abundance and in head size, as well as drastic mobility impairments and induction of apoptosis in the brain, thus phenocopying the disease in mammals. The transcriptomic analysis of NPCs isolated from whole larvae revealed that ZIKV downregulated the expression of the glutamate transporter *vglut1*, which was associated with an altered network of glutamatergic neurons in the brain. In contrast, ZIKV infection increased the expression of regulators of cell survival, apoptosis and differentiation, such as *pim2*, *cbx7a*, as well as components of the activator protein 1 (AP-1) family members (*jun*, *junB*, *fosab*). Importantly, the sole expression of ZIKV protein NS4A in the larvae recapitulated the morphological defects observed in infected animals. By mimicking several phenotypes and symptoms in humans caused by ZIKV, this innovative animal model provides a unique and unprecedented access to the ZIKV-infected brain of vertebrates to further investigate the host and viral determinants of ZIKV neuropathogenesis *in vivo*. Given the efficacy of a known anti-ZIKV drug in this system, it provides a suitable platform for rapidly testing antiviral molecules *in vivo*.

## Results

### Zika virus replicates efficiently in zebrafish larvae and induces morphological defects

**Fig 1:**
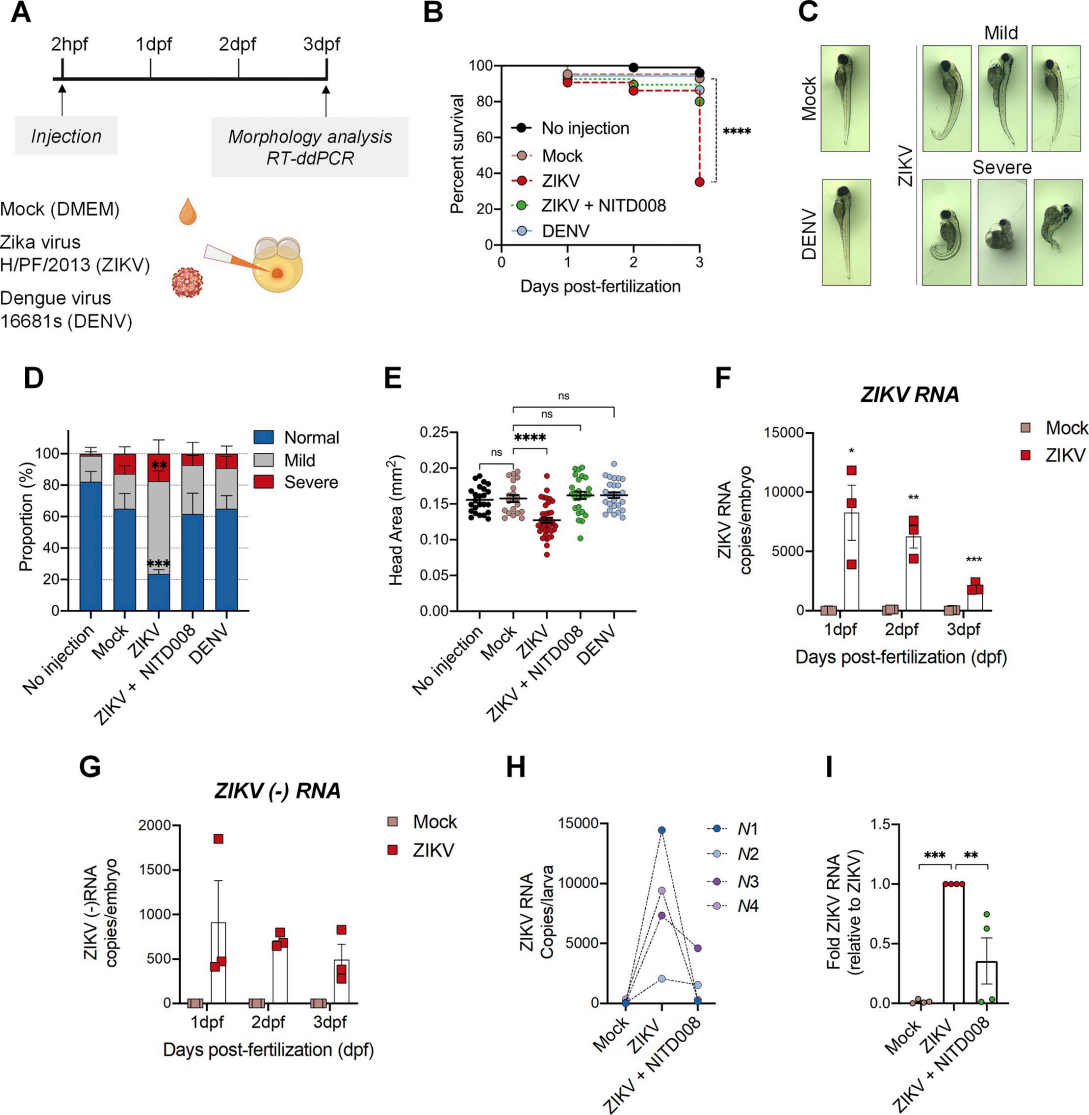
Zika virus replicates efficiently in the zebrafish model and induces morphological defects. (A) Schematic experimental design. Cell medium (mock), DENV viral particles (serotype 2, strain 16681s) or ZIKV viral particles (strain H/PF/2013) were microinjected in the zebrafish yolk at 2 hours post-fertilization (hpf). This schematic was created with BioRender.com. (B) Survival curve over 3 days post-fertilization (dpf) of mock-infected (*n* = 84), ZIKV-infected (*n* = 104), ZIKV-infected and treated with 100 μM NITD008 for the whole period (*n* = 94), and DENV-infected (*n* = 89) larvae. (*N* = 3) **** P ≤ 0.0001. Log-rank test. (C) Representative pictures of microinjected larvae at 3 dpf. ZIKV infection induced both mild and severe developmental phenotypes. (D) Quantification of the proportion of larvae with the different morphological phenotypes illustrated in (C). Visual assessment of zebrafish morphology was done based on the criteria listed in S1A Fig. (No injection, *n* = 64; Mock, *n* = 54; ZIKV, *n* = 67; ZIKV+NITD008 (100 μM), *n* = 62; DENV, *n* = 55. *N* = 3). Data are shown as means ± SEM. *** P ≤ 0.001; ** P ≤ 0.01; 2-way ANOVA. (E) Head size at 3 dpf of the larvae from (D) (No injection, *n* = 24; Mock, *n* = 20; ZIKV, *n* = 38; ZIKV+NITD008 (100 μM), *n* = 25; DENV, *n* = 26. *N* = 3). Data are shown as means ± SEM. **** P ≤ 0.0001; ns = not significant; one-way ANOVA. (F-G) Total ZIKV RNA (F) and ZIKV negative strand (-) RNA (G) levels in whole larvae pools (6–15 larvae) at 1, 2 and 3 dpf were determined using ddPCR. Absolute RNA copies per fish per day post-fertilization are shown. *N* = 3. Data are means ± SEM. *** P ≤ 0.001; ** P ≤ 0.01; * P ≤ 0.05; Student’s t-test for each day. (H-I) Treatment of ZIKV-infected fish with 100 μM NITD008 decreased the viral load. Viral RNA copies (normalized to the number of larvae) are shown for each independent experiment in (H). Dash lines indicate results from the same independent experiment. In (I), data were normalized to the corresponding ZIKV value for each experiment. Mock, *n* = 40; ZIKV, *n* = 40; ZIKV+NITD (100 μM), *n* = 40. *N* = 4. Data are shown as means ± SEM. *** P ≤ 0.001; ** P ≤ 0.01; one-way ANOVA. *n* represents the number of fish; *N* represents the number of independent experimental repeats.

To establish an alternative *in vivo* animal model for ZIKV infection, we microinjected ~100 infectious virus particles (PFUs) of the ZIKV H/PF/2013 strain (epidemiologically linked to CZS) in the yolk of zebrafish embryos within the two first hours following fertilization to be consistent with an infection during early pregnancy (Fig 1A). Embryos injected with DMEM (vehicle, mock) were used as reference controls. At 3 days post-fertilization (dpf), zebrafish larvae were assessed for changes in viability and morphology according to the criteria shown in S1A Fig. We observed that 3 dpf ZIKV-infected larvae exhibited a marked decrease of approximatively 65% in the survival rate compared to controls (Fig 1B). ZIKV-infected larvae also showed drastic developmental defects at 3 dpf (Fig 1C and 1D). More specifically, ~80% of the living larvae exhibited mild or severe morphological defects, ranging from curved and shorter tail, edema to ovoid morphology. Importantly, a significant reduction of 20% in the head area was noted following infection with ZIKV compared to the controls (Figs 1E and S1B), which is reminiscent of newborn microcephaly in humans. Remarkably, injection of DENV, a non-neurovirulent orthoflavivirus did not induce any apparent morphological defects particles (except pericardial edema in some larvae). Moreover, in contrast to ZIKV, no differences in viability or morphology were observed between mock- and DENV-infected larvae (Figs 1B–1E). Following total RNA extraction from whole injected fish at 1 dpf, 2 dpf and 3 dpf, and reverse transcription droplet digital PCR (RT-ddPCR), we could readily detect total vRNA of both ZIKV and DENV (Figs 1F and S2A). Most importantly, the negative strand of the viral RNA (ZIKV (-) RNA), an intermediate RNA species produced by NS5 RNA polymerase during RNA replication, was also detected at 1, 2 and 3 dpf using strand-specific RT-ddPCR (Fig 1G). Altogether, these data support that ZIKV replicates in zebrafish embryo/larvae and further suggest that the defects induced by ZIKV injection in zebrafish larvae are specific to this virus.

Importantly, toward demonstrating that ZIKV replication in zebrafish causes these morphological defects, NITD008 (final concentration 100 μM), a nucleoside analog which inhibits ZIKV NS5 RNA polymerase activity and thus, viral replication [37], was added to the fish water at 4 hours post-infection. While viral RNA was detected at 3 dpf, its levels were reduced in larvae treated with NITD008, confirming that the virus replicates in the animal (Fig 1H–1I). Most importantly, NITD008-treated infected larvae had similar survival rate and morphology to uninfected fish (Fig 1B–1E), supporting that the observed defects were solely due to ZIKV replication.

Surprisingly, RT-qPCR with total RNA extracted from entire infected larvae did not detect any significant induction of mRNAs encoding interferons (*ifnΦ1*, *ifnΦ3*), interferon-stimulated genes *rig-I*, *mx* and *viperin*, and proinflammatory cytokine *TNFa* at either 1 or 3 dpf when ZIKV was injected (S2B and S2C Fig). Only a modest 2.6-fold increase of *isg15* mRNA levels was detected at 3 dpf. Comparable expression data were observed upon DENV injection (S2D and S2E Fig). We have confirmed that the larvae from our wildtype fish line used for infection possess a competent innate immune system since the injection of poly(I:C) (a synthetic double-stranded RNA mimicking viral RNA) at 2 hours post-fertilization resulted in a readily detectable induction of the tested immune genes at 1 day post-fertilization (S2F–S2H Fig). It is noteworthy that such treatment resulted in a high mortality rate compared to mock-injected animals, which correlated with an exacerbated interferon response in the dead animal. In parallel, we took advantage of another ZIKV isolate, namely HS-2015-BA-01 (referred to herein as Brazil 2015) [38,39], which we confirmed to be more cytopathic than ZIKV H/PF/2013 in human cell culture (S3A Fig) with faster replication kinetics (S3B Fig, 24 hours post-infection) as reported before [38,40]. Very strikingly, when we infected zebrafish embryos with this highly pathogenic ZIKV strain, the animals exhibited aberrant morphology and organ development at 1dpf compared to control embryos (S3C Fig), and died prematurely before 2 dpf (S3D Fig). At 1 dpf, we could readily detect infectious viral particles from these dissociated embryos in plaque assays (S3E Fig) in contrast to H/PF/2013 for which viral titers were detectable, yet very close to the limit of detection. This correlated with the detection of *ifnΦ3*, *TNFa*, *mx*, *isg15* and *viperin* inductions (S3F Fig). Consistent with the poly(I:C)-related data, this suggests that when ZIKV induces innate immunity, the animals do not develop and die, implying that only infected animals with no or low interferon-stimulated genes/interferon expression can survive. Overall, these data along with the fact that DENV injection did not induce any major defects, strongly indicate that morphological defects observed at 3 dpf are specifically due ZIKV replication and were not indirectly caused by innate immunity-dependent inflammatory responses.

**Fig 2:**
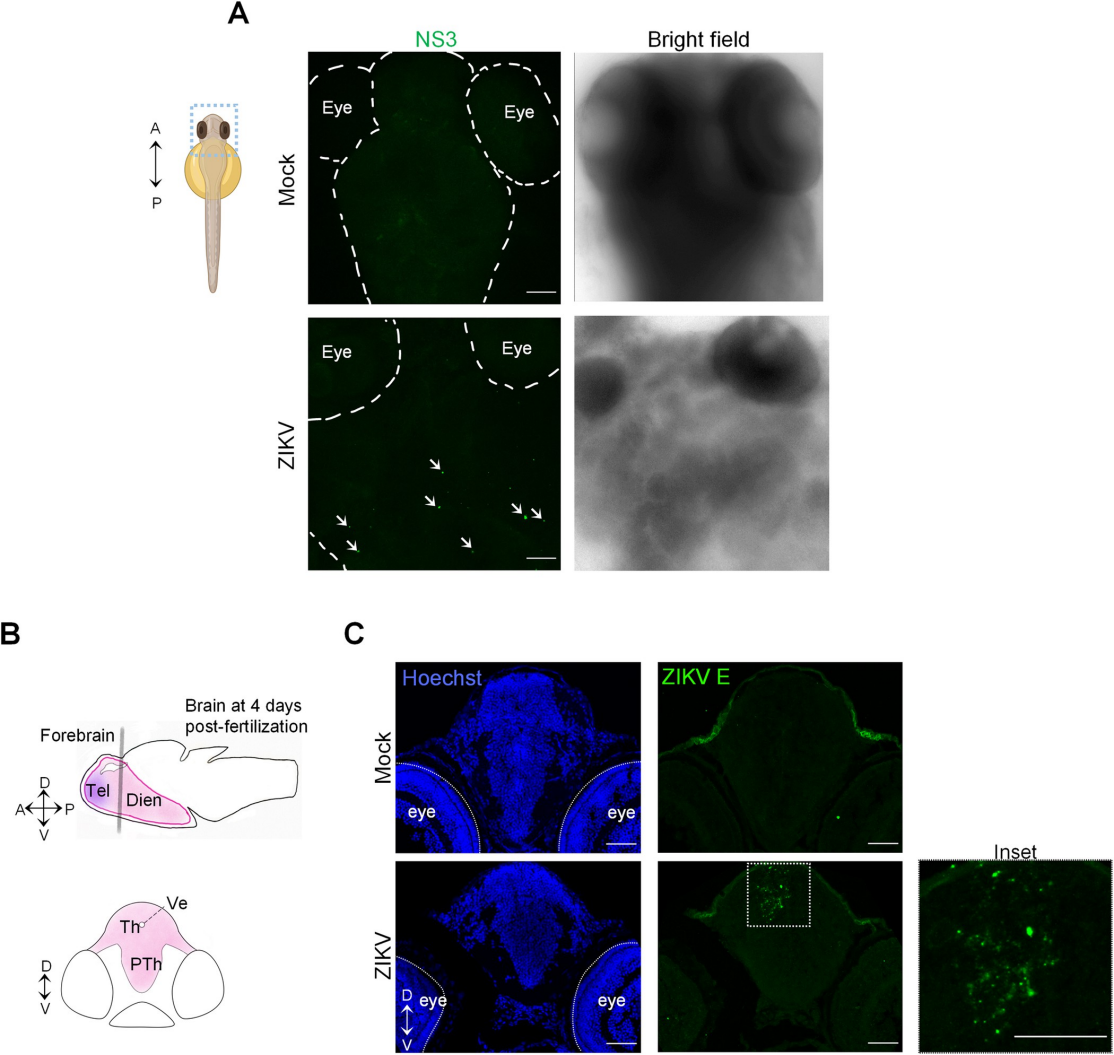
ZIKV injection results in viral protein accumulation in larval brain. (A) At 3 dpf, mock- and ZIKV-infected larvae were fixed and subjected to whole mount immunostaining with anti-NS3 antibodies. Representative pictures of 4 imaged samples are shown. The arrows indicate specific ZIKV NS3 signals. The schematic representation of the zebrafish larva illustrating the region of interest was created with BioRender.com. A = anterior; P = posterior. (B) Schematic representation of a zebrafish brain at 4 days post-fertilization. The forebrain is shown. The gray line represents the localization of the transverse section. Th and PTh are areas of the diencephalon, a division of the forebrain. Dien = diencephalon; Tel = telencephalon; Th = thalamus; PTh = prethalamus; Ve = ventricle; A = anterior; P = posterior; D = dorsal; V = ventral. (C) Transverse brain cryo-sections of 4 days post-fertilization mock-injected or ZIKV-injected larvae are shown. Sections were stained with anti-ZIKV E antibody (green) and analyzed by confocal microscopy. Nuclei were labeled with Hoechst. Images are maximal intensity projections. Scale bars = 50 μm.

To test whether ZIKV is neurotropic in developing zebrafish, we first performed wholemount immunostaining on larvae at 3 dpf and imaged the heads using confocal microscopy. Since the embryos injected with ZIKV Brazil 2015 did not develop past 1 dpf, we used H/PF/2013 as a study virus. Viral protein NS3 was detected in the brain of ZIKV-injected animals (Fig 2A). We further examined the ZIKV neurotropism at 4 dpf at the histological level on larval brain cryosections using a panflaviviral anti-envelope (E) antibody. Infection foci were specifically detected in the developing forebrain and midbrain of ZIKV-infected animals compared to control animal with high signal intensity in the thalamus of the diencephalon, an area surrounding the brain ventricle in the forebrain (Fig 2B and 2C). This indicates that ZIKV is neurotropic in the larva even if the injection was performed at a time at which cells were pluripotent [41].

Taken together, our findings unambiguously demonstrate that zebrafish larva is permissive to ZIKV which replicates in the developing brain, inducing phenotypes highly reminiscent of those observed in infected human fetuses. This strongly suggests that the observed ZIKV-induced morphological and head defects are caused by ZIKV brain neurovirulence as in mammals.

### ZIKV infection causes mobility defects

While it is clear that ZIKV targets the central nervous system (CNS) as in human, we have further investigated to which extent the zebrafish model mirrors the human disease in terms of neurovirulence and severity of the symptoms. Previous epidemiological studies showed that motor impairment is associated to CZS in Human [42,43]. Of note, locomotor activities in zebrafish are closely linked to brain function integrity, to visual development, to muscle activity, and more importantly to nervous system development [44–47]. Therefore, we hypothesized that motor activity can be used as a readout of nervous system development and brain abnormalities in zebrafish. To challenge this hypothesis, we performed a touch-elicited escape behavioral assay using live animal imaging at 2 and 3 dpf in ZIKV-infected fish and controls. Most ZIKV-injected animals with mild morphological defects were unable to flee at 2 dpf, displaying abnormal circular swimming patterns upon stimulation when compared to controls (Video S1). At 3 dpf, very little movement, if any was observed for the infected larvae (Video S2). Very strikingly, NITD008 treatment seemingly reverted these swimming defects (Videos S1-2). This observation is consistent with the inhibition of ZIKV replication following treatment with this drug (Fig 1H and 1I).

**Fig 3:**
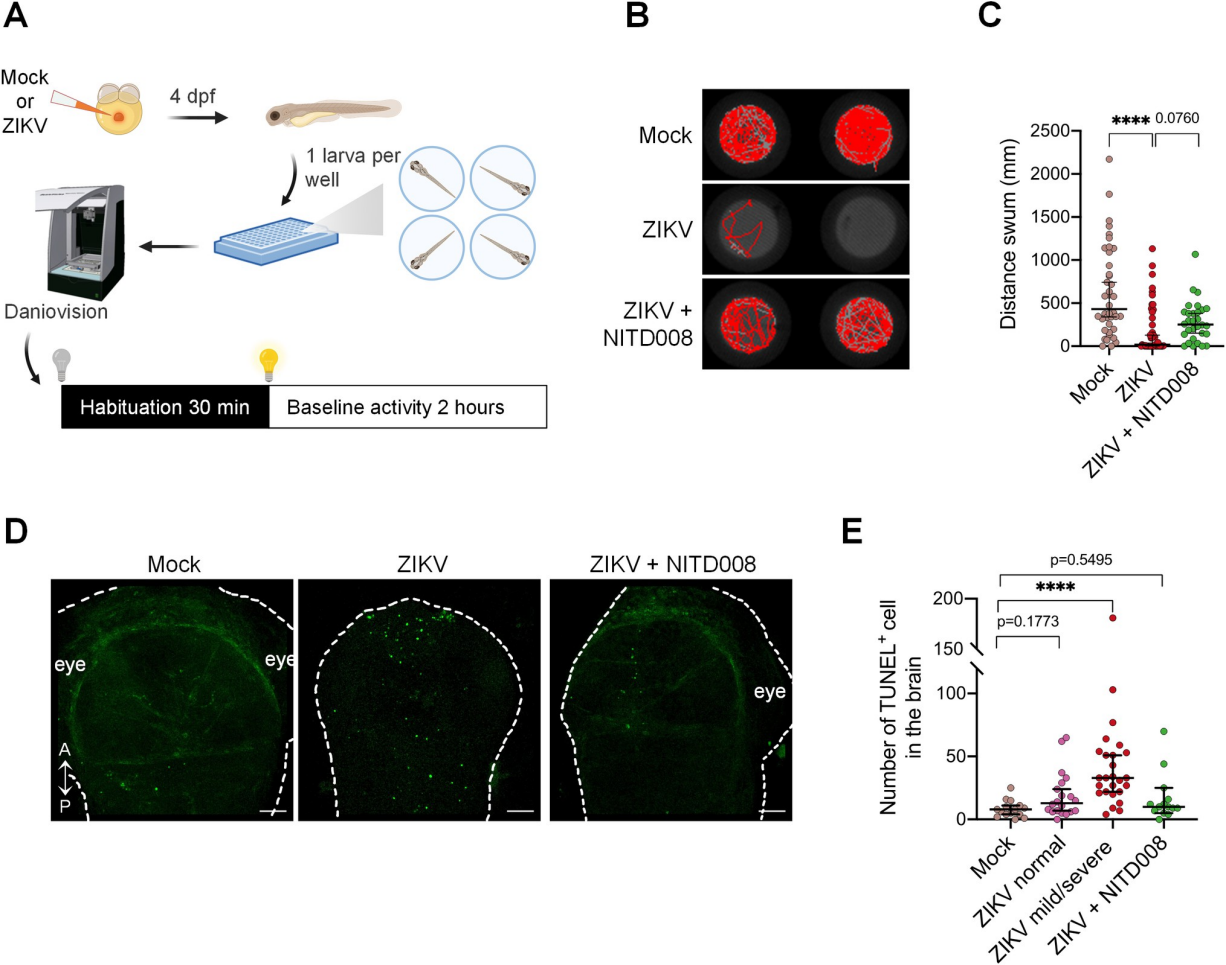
Locomotor defects and brain cell death in developing zebrafish following ZIKV infection. (A) Schematic experimental setup and behavioral analysis (created with BioRender.com). (B) Representative swimming tracks of control (mock), ZIKV-infected (ZIKV), and ZIKV-infected and NITD008-treated (ZIKV+NITD008 (100 μM)) larvae at 4 days post-fertilization. (C) The distance moved by the larvae was assessed using the DanioVision device (Mock, *n* = 40; ZIKV, *n* = 45; ZIKV+NITD008 (100 μM), *n* = 30. *N* = 3). Data are shown as median ± 95 CI. **** P ≤ 0.0001; Kruskal-Wallis test. (D) TUNEL staining at 2 days post-fertilization showing cell death in the developing brain following injection in zebrafish embryos. A = anterior; P = posterior. Scale bars = 50 μm. (E) The number of TUNEL+ cells was quantified (Mock, *n* = 15, *N* = 3; ZIKV normal, *n* = 20, *N* = 3; ZIKV mild/severe, *n* = 26, *N* = 3; ZIKV+NITD008 (100 μM), *n* = 14, *N* = 2). Data are shown as median ± 95 CI. **** P ≤ 0.0001; Kruskal-Wallis test. *n* indicates the number of fish; *N* represents the number of independent experimental repeats.

To rigorously assess the impact of ZIKV infection on the motor system, we used the Daniovision, an automated observation chamber which allows the quantitative analysis of motor behaviors (Fig 3A). At 4 days post-fertilization, ZIKV-infected larvae displayed aberrant swimming behavior. Indeed, zebrafish infected larvae had little to no movement (Fig 3B). Using this technique, we showed that the distance swum following infection was severely reduced by ~25-fold when compared to control larvae (Fig 3C), consistent with an increase in immobility time (S1C and S1D Fig). In accordance with live imaging observations, NITD008 partially restored the swimming capacity (Fig 3B and 3C). Moreover, DENV injection did not induce any mobility defects (S1C and S1D Fig), suggesting that the phenotypes are specific to ZIKV replication.

### ZIKV induces cell death in the developing brain of zebrafish

Earlier studies have shown that ZIKV infection leads to cellular death *in vivo* and *in vitro* [5,6]. Considering ZIKV neurotropism, the reduction in head area and the locomotor defects in our zebrafish larva model, we sought to investigate further the extent of neurovirulence by evaluating ZIKV-induced apoptosis in the developing brain. At 2 days post-fertilization, whole fixed larvae were subjected to terminal deoxynucleotidyl transferase-mediated dUTP nick end-labeling (TUNEL) assays to detect apoptotic cells by confocal microscopy. Subsequent quantification of the TUNEL-positive cells revealed an overall 3-fold increase in the number of apoptotic cells in the developing brain following viral infection, as compared to the control (Fig 3D and 3E). More precisely, ZIKV-injected larvae displaying mild or severe morphology defects exhibited a ~4-fold increase in brain cell death compared to the control (Fig 3E). Notably, even ZIKV-injected larvae with seemingly normal morphology displayed an increased number of apoptotic cells when compared to the control group, although the difference was not statistically significant (Kruskal-Wallis test, p-value = 0.1773). Interestingly, ZIKV-injected larvae treated with NITD008 had similar cell death level as the control (Fig 3D and 3E). These results demonstrate that ZIKV replication induces cell death in the developing brain.

### ZIKV infects neural progenitor cells and induces their depletion in zebrafish developing brain

**Fig 4:**
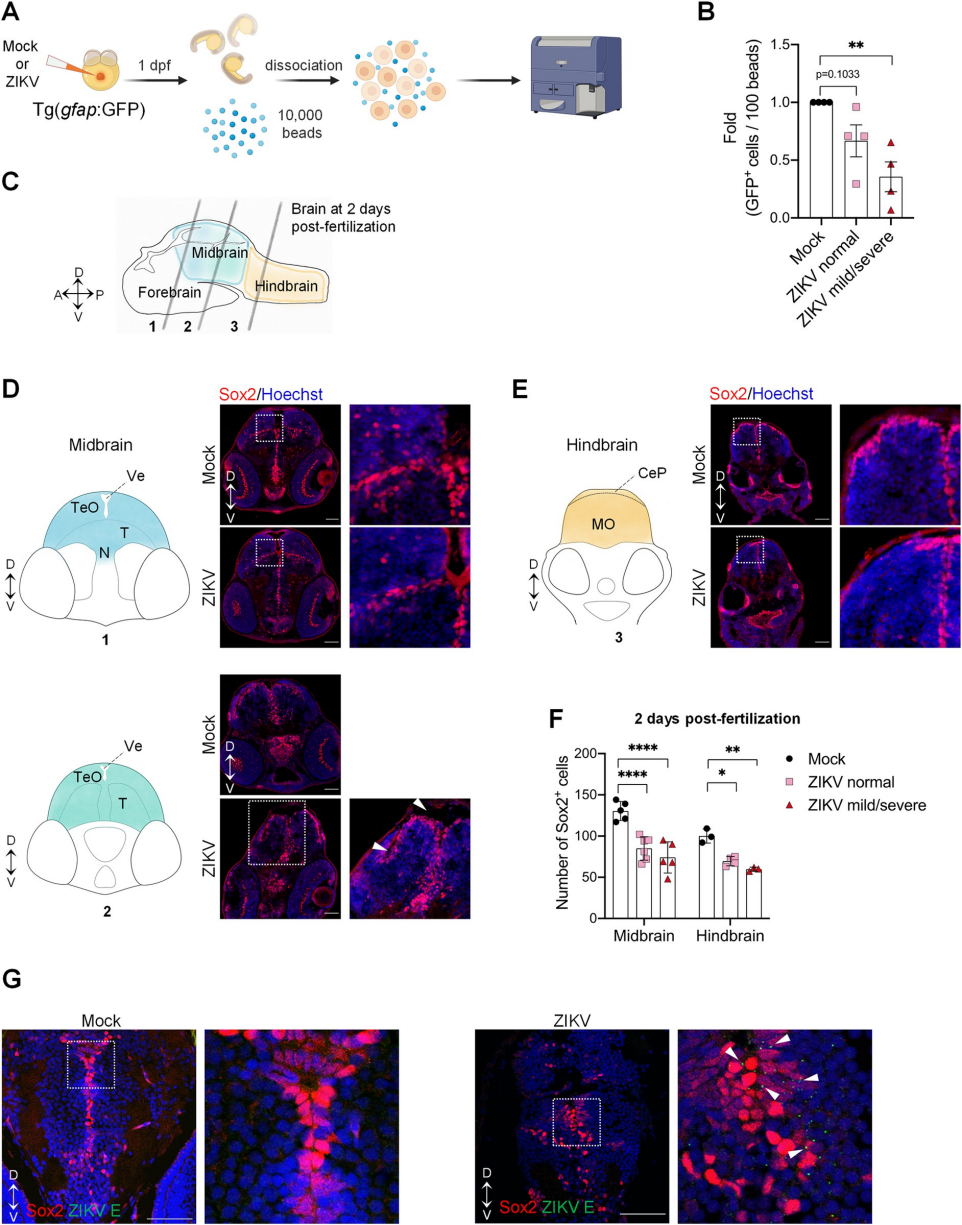
Zika virus targets neural progenitor cells and induces neuropathogenesis in zebrafish larvae. (A) Schematic experimental design (created with BioRender.com). At 1 day post-fertilization, ZIKV infected or uninfected whole transgenic Tg(*gfap*:GFP) embryos were dissociated in the presence of 10,000 fluorescent normalizing beads. Single cells and beads were counted by flow cytometry. (B) Relative abundance of GFP^+^ cells counted per 100 beads (*N* = 4). Data are shown as means ± SEM. ** P ≤ 0.01; one-way ANOVA. (C) Schematic representation of a zebrafish developing brain at 2 days post-fertilization showing the three areas of the brain: forebrain, midbrain, and hindbrain. The gray lines represent the localization of the transverse sections. D = dorsal; V = ventral; A = anterior; P = posterior. (D-F) Number of neural progenitor cells (Sox2^+^ cells) in the midbrain (D) and the hindbrain (E) of 2 days post-fertilization mock-injected or ZIKV-injected embryos. TeO, N, and T are areas of the midbrain while MO is an area of the hindbrain. TeO = tectum opticum; T = midbrain tegmentum; N = region of the nucleus of medial longitudinal fascicle; Ve = ventricle; MO = medulla oblongata; CeP: Cerebellar plate. D = dorsal; V = ventral. Data are shown as means ± SEM. **** P ≤ 0.0001; ** P ≤ 0.01; * P ≤ 0.05; Two-way ANOVA. Scale bars = 50 μm. White arrowheads indicate altered midbrain ventricles. (G) Confocal microscopy of brain section from mock-injected and ZIKV-injected embryos at 4 dpf. Cells were co-immunostained with anti-Sox2 and anti-ZIKV E. Cell nuclei were counterstained with Hoechst. Scale bars = 50 μm. White arrowheads indicate E cytoplasmic foci next to Sox2-positive nuclei. These images are representative of 3 analyzed brains. *N* represents the number of independent experimental repeats.

Among other cell types, NPCs were described to be a major target of ZIKV in human fetal brains. Thus, we investigated the impact of ZIKV infection on NPC abundance and their distribution in the brain. First, we took advantage of the transgenic line Tg(*gfap*:GFP), which allows visualization and quantification of NPCs at 1 day post-fertilization. In this transgenic line, the native (*i*.*e*. non-fused) green fluorescent protein (GFP) is expressed under the transcriptional control of the glial fibrillary acidic protein (*gfap*) promoter, a marker of NPC at this early time point of brain development [48,49]. First, we confirmed that ZIKV injection in these transgenic embryos recapitulates the morphological defects observed in wildtype larvae (S4A Fig). Next, Tg(*gfap*:GFP) embryos were infected with ZIKV or left uninfected and 24 hours post-fertilization, *i*.*e*., shortly after neurogenesis induction [50], whole embryos were dissociated into single cells. GFP-positive cells were quantified by flow cytometry in the presence of fluorescent beads, allowing to normalize for dissociation efficiency, and unbiased cell counting as described before (Fig 4A) [51]. Reverse transcription PCR (RT-qPCR) on sorted GFP^+^ cells confirmed high endogenous expression of *nestin* and *gfap* mRNAs, hallmarks of NPCs at 1 dpf, when compared to GFP^-^ cells (S4D Fig), confirming selective expression of GFP in the neural progenitor cells [52,53]. Strikingly, ZIKV infection induced a decrease in the abundance of GFP^+^ cells, *i*.*e*., NPCs, compared to the control (Fig 4B). Particularly, we observed a mean 64.4%-fold decrease of NPC levels (one-way ANOVA; p ≤ 0.01) when the phenotype was mild or severe (Fig 4B). In addition, we investigated another transgenic line, that is Tg(*nestin*:GFP), allowing the detection of neuronal progenitors, a subclass of NPCs. Similar to wildtype line larvae, ZIKV injection induced morphological defect in this line (S4B Fig). Consistently with the results obtained with Tg(*gfap*:GFP), the number of neuronal progenitor cells (GFP^+^) was markedly decreased in Tg(*nestin*:GFP) (S4C Fig). This depletion of NPCs was not attributed to a non-specific developmental delay since the injection of ZIKV into Tg(*flk1*:EGFP) embryos (in which EGFP is specifically expressed in endothelial cells) [54] did not significantly change the abundance of EGFP-positive cells at 1 dpf as assessed by flow cytometry although ZIKV induced morphological defects in this transgenic line (S4F and S4G Fig). These data unambiguously demonstrate a specific loss of NPCs in the whole embryo following ZIKV-infection.

We aimed to gain more insight into NPC distribution and abundance in different brain regions following ZIKV infection. Brain cryosections of ZIKV-infected fish and controls at 2 and 4 dpf were immunolabeled for Sox2, another marker for neural progenitor cells which as a transcription factor, localizes in the nucleus (Figs 4C–4F and S5) [50]. In agreement with earlier reports, multiple layers of Sox2^*+*^ cells could be identified in the tectum opticum of control animals (Fig 4D) at 2 dpf, while ZIKV-infected embryos displayed a significantly thinner layer (Fig 4D top insets) [55]. At this time, Sox2^+^ cells were also located in periventricular zones, which are in direct contact with the midbrain and hindbrain ventricles. More precisely, they were lining the walls of the midbrain midline ventricle (Fig 4D bottom) and were in the ventricular surface of the cerebellar plate, and in medulla oblongata (Fig 4E). In control brain, NPCs are distributed along the ventricular surfaces, following a dorsomedial-dorsolateral distribution as previously described [55]. Compared to mock, Sox2^+^ cells in ZIKV-infected embryo were distributed to a lesser extent on the lateral sides of the midbrain and hindbrain, suggesting a defect in NPCs migration/positioning (Fig 4D and 4E). Quantification of Sox2^+^ cells in the midbrain at 2 dpf revealed a significant decrease in the number of NPCs of ZIKV-infected fish displaying both normal, and mild/severe phenotypes (Fig 4F). Consistently, depletion of NPCs was also observed in the midbrain at 4 dpf (S5A, S5B and S5D Fig), and in the hindbrain at 2 and 4 dpf (Figs 4E, 4F, S5C and S5D). This demonstrates that ZIKV infection specifically targets the pools of NPCs, reducing their number and density in different parts of the developing brain and interfering with their positioning. Of note, brains from ZIKV-infected larvae displayed a dilated ventricle compared to control (Fig 4D, bottom, white arrowheads), resembling the ventriculomegaly and hydrocephaly observed in ZIKV-infected human newborns [56,57].

In order to correlate this impairment of NPCs with ZIKV infection, 4 dpf brain sections were co-stained with antibodies directed against Sox2 and ZIKV E protein. E cytoplasmic foci were primarily detected juxtaposed to Sox2-positive nuclei (Fig 4G, white arrowheads), supporting that a subpopulation of ZIKV-infected cells comprises NPCs. Infected NPCs mostly accumulated in periventricular regions rich in Sox2^*+*^ cells (Fig 4G). Altogether, these observations demonstrate that ZIKV infection of zebrafish at early stages of development closely resembles the pathophysiology of human infections upon vertical transmission with respect to the physiology, neurodevelopmental sequelae and specific perturbation of the NPCs pool in terms of abundance and distribution.

### ZIKV infection modulates the expression of genes involved in cellular proliferation, differentiation, and apoptosis in NPCs

To identify the specific molecular mechanisms and viral targets underlying ZIKV-induced impairment of NPC abundance, we investigated the impact of ZIKV infection on the transcriptional landscape of NPCs during development *in vivo*. NPCs were isolated from ZIKV-infected and control Tg(*nestin*:GFP) embryos at 1 dpf using fluorescence-activated cell sorting (FACS). Transcriptomic analysis of NPCs was performed at this stage (*i*.*e*. 24 hours post-fertilization), when proliferation and differentiation are well established. We confirmed that NPC-specific endogenous *nestin* and *gfap* mRNAs were enriched in isolated GFP^+^ cells, compared to GFP^-^ cells (S4E Fig). RNA sequencing of NPCs isolated from control and ZIKV-infected embryos revealed significant transcriptional modulation of 199 genes in NPCs upon ZIKV infection, with 104 and 95 genes significantly up- and down-regulated, respectively (S1 Table; P<0.01; Log_2_FoldChange > 0.7 or < -0.7).

**Fig 5:**
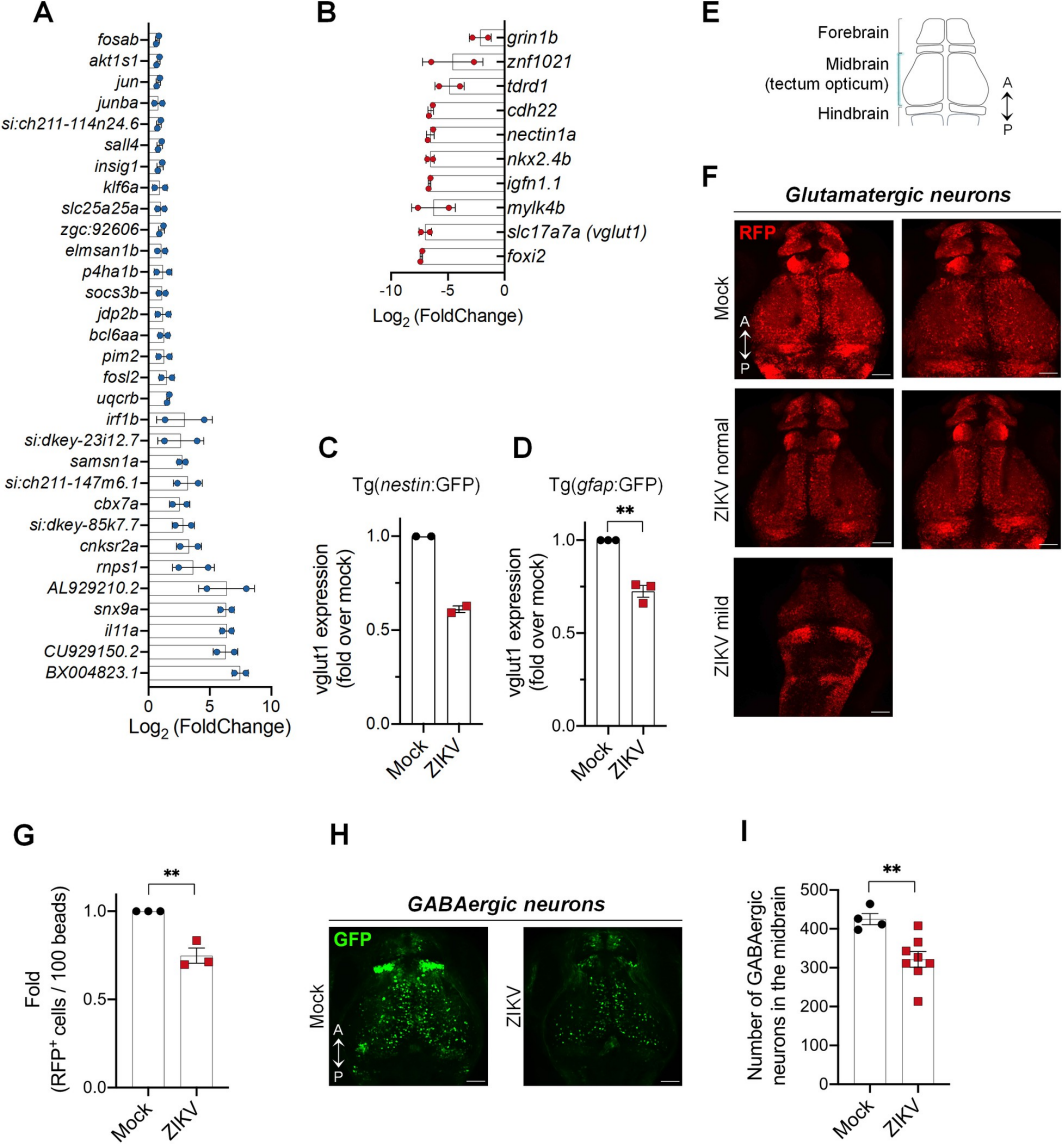
ZIKV infection dysregulates the NPC transcriptome and disrupts the glutamatergic and GABAergic neuronal networks. (A-B) Tg(*nestin*:GFP) embryos were infected with ZIKV or mock-injected. At 1 dpf, NPCs (*i*.*e*., GFP^+^ cells) were isolated from whole larvae using FACS. NPC transcriptome was analyzed using RNA sequencing. mRNA expression levels were compared to that of NPCs isolated from uninfected control larvae. The most significantly upregulated (A) and downregulated (B) genes are shown (*p* < 0.01) (Mock, *n* = 30; ZIKV, *n* = 31. *N* = 2). (C-D) *vglut1* gene expression in NPCs isolated from infected larvae, compared to uninfected control larvae, was analyzed by ddPCR. Two transgenic fish lines, Tg(*nestin*:GFP) (C) and Tg(*gfap*:GFP) (D), were used. (E) Schematic representation of the dorsal view of the zebrafish brain at 3 dpf showing the three areas: forebrain, midbrain, and hindbrain. A = anterior; P = posterior. (F) Representative images of the glutamatergic neuronal network (RFP^+^ cells) in the whole brain of control and ZIKV-injected at 3 dpf. Tg(*dlx5a/6a*:GFP;*vglut2*:RFP) larval brains were imaged by confocal microcopy. *N* = 3. Scale bars = 50 μm. (G) Control and ZIKV-injected transgenic larvae Tg*(dlx5a/6a*:GFP;*vglut2*:RFP) expressing RFP under the control of the *vglut2* promoter (*i*.*e*., specifically in glutamatergic neurons) were dissociated at 3 dpf in the presence of 10,000 fluorescent normalizing beads. Single cells and beads were counted by flow cytometry. Number of RFP^+^ cells per 100 beads were counted (Mock, *n* = 18; ZIKV, *n* = 18; *N* = 3). The relative abundances are shown as means ± SEM. ** P ≤ 0.01; Student’s t-test. (H-I) Representative images of GABAergic neurons (GFP^+^ cells) in the whole brain of control and ZIKV-injected at 3 dpf. (I) Quantification of the number of GFP-positive cells in the midbrain from images of (H) by manual counting (Mock, *n* = 4; ZIKV, *n* = 8; *N* = 2). Data are shown as means ± SEM. ** P ≤ 0.01; Student’s t-test. Scale bars = 50 μm. *n* indicates the number of fish; *N* represents the number of independent experimental repeats.

Interestingly, mRNAs encoding cell proliferation and apoptotic regulator factors such as *pim2* and AP-1 transcription factor members *junba*, *jun and fosab* were increased. *Chromobox homolog 7a (cbx7a)* expression, which inhibits differentiation, axon growth and axon regeneration was also increased by 6.7-fold (p-value = 4.59 x 10^−7^, Fig 5A) [58,59]. These results suggest a ZIKV-induced rewiring of zebrafish NPC survival and neuronal maturation networks, consistent with those reported for mammalian NPCs [11].

Notably, one of the most affected genes was *vglut1*, displaying a 130-fold reduced expression upon ZIKV infection (p-value = 2.39 x 10^−5^, Fig 5B). RT-ddPCR analysis of NPCs isolated from infected Tg(*nestin*:GFP) and Tg(*gfap*:GFP) embryos showed a decreased expression of *vglut1*, thus confirming the expression profile of *vglut1* in the RNA sequencing results (Fig 5C and 5D). *Vglut1* mRNA encodes the glutamate transporter, which is required for the release of this neurotransmitter by presynaptic excitatory neurons. It is thus a *bona fide* marker of glutamatergic neurons. Additionally, the abundance of *glutamate receptor ionotropic NMDA 1b* (*grin1b*) mRNA was also decreased (Fig 5B). The protein encoded by this gene is a subunit of the NMDA glutamate receptor. *GRIN1* mutations, and more broadly defects in the functionality of these glutamatergic neurons, are typically associated with cognitive impairments, epilepsy, microcephaly, muscular tone abnormalities, and behavior issues [60–63]. Interestingly, such impairments are a sequelae of children born with the congenital Zika syndrome [64,65]. To test whether *vglut1* and *grin1b* mRNA level decreases correlate with a glutamatergic network alteration, we characterized this mature neuron population at 3 dpf (a time point at which the mature neuronal network is established) using the Tg(*dlx5a/6a*:GFP;*vglut2*:RFP) fish line, in which glutamatergic neurons express red fluorescent protein (RFP) while GFP is produced in GABAergic neurons. Whole brain imaging identified marked reduction in RFP intensity signal in all parts of the brain *i*.*e*., forebrain, midbrain and hindbrain following ZIKV infection (Fig 5E and 5F). Further quantification of RFP^+^ cells (*e*.*g*., glutamatergic neurons) from dissociated larvae by flow cytometry confirmed a significant reduction of glutamatergic neurons at 3 dpf in ZIKV infected larval brains compared to controls (Fig 5G). Altogether, these data suggest that ZIKV infection disrupts the excitatory glutamatergic network in the developing vertebrate model.

In addition to cognitive impairments, epilepsy/seizure are a frequent occurrence in children with CZS [66,67]. Recently, an alteration of GABAergic interneurons was shown to be associated with hyperactivity in zebrafish, γ-Aminobutyric acid GABA being the primary inhibitory neurotransmitter in the CNS [35]. The abundance of GABAergic neurons (*i*.*e*., number of GFP^+^ cells) was significantly reduced in Tg(*dlx5a/6a*:GFP;*vglut2*:RFP) larvae following ZIKV infection compared to control (Fig 5H and 5I), although it is noteworthy that no change in the expression of *dlx* genes (*dlx1a*, *dlx2a*, *dlx5a*, and *dlx6a*) involved in the specification of GABAergic neurons was noted in our NPC transcriptomic analysis (S1 Table). Collectively, our results suggest that the cognitive impairment and neurodevelopmental disorder induced by ZIKV are partly due to loss of glutamatergic and GABAergic neurons, likely caused by an alteration of neurogenesis.

### NS4A is a major viral determinant of ZIKV neuropathogenesis

**Fig 6:**
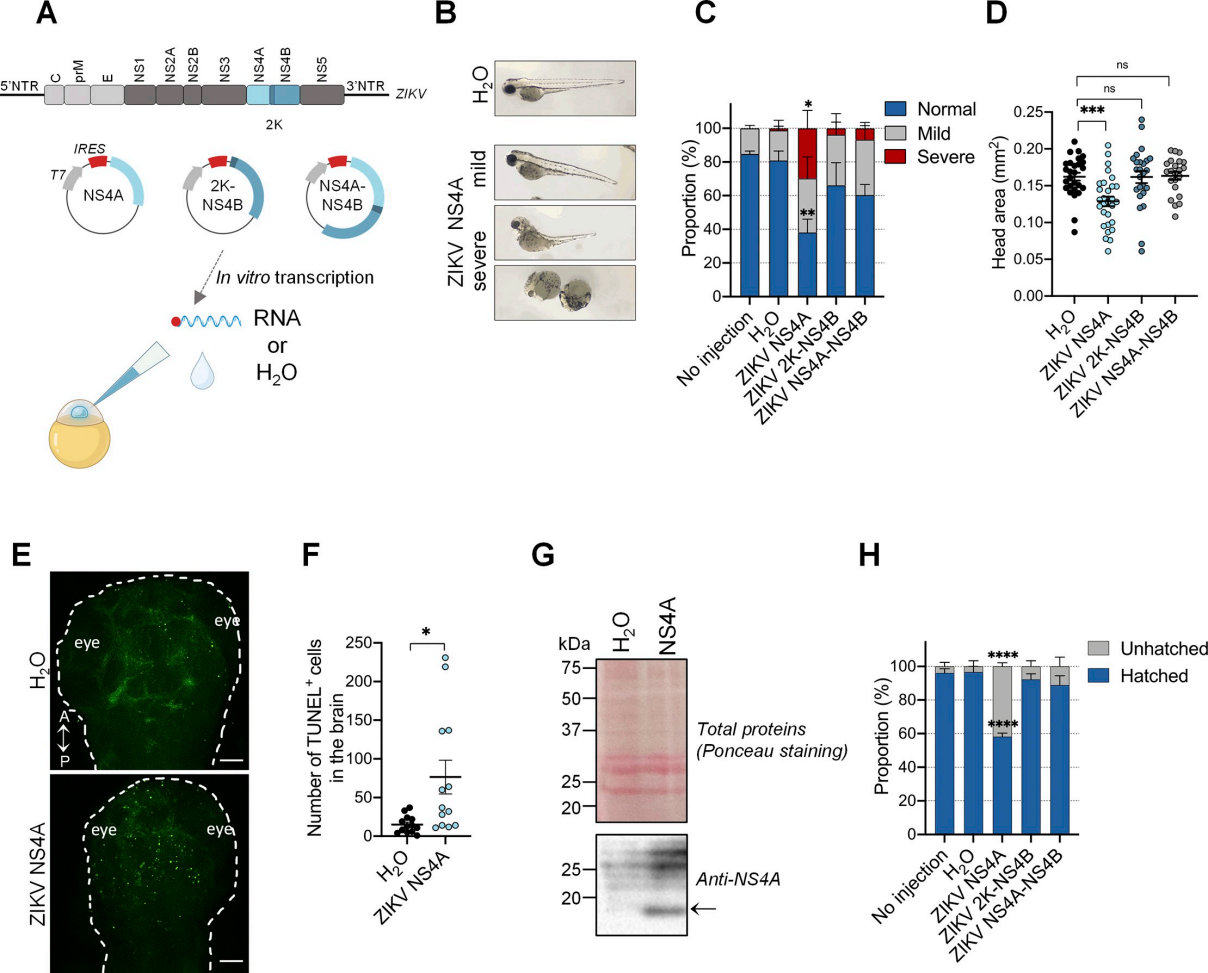
ZIKV NS4A is a major viral determinant in ZIKV pathogenesis *in vivo*. (A) Schematic experimental design (created with BioRender.com). ZIKV genome is represented. *In vitro* transcription was performed on plasmid coding for NS4A, 2K-NS4B, and NS4A-NS4B. NTR = non-translated region; IRES = internal ribosomal entry site. *In vitro* transcribed NS4A, 2K-NS4B, NS4A-NS4B RNAs were injected at one-cell stage at 1 hour post-fertilization. H_2_O was used as the reference control. (B) Representative pictures of microinjected larvae at 3 days post-fertilization. NS4A expression induced both mild and severe developmental phenotypes. (C) Proportion of larvae with different phenotypes at 3 days post-fertilization (No injection, *n* = 68; H_2_O, *n* = 53; ZIKV NS4A, *n* = 62; ZIKV 2K-NS4B, *n* = 62; ZIKV NS4A-NS4B, *n* = 63. *N* = 3). Data are shown as means ± SEM. **** P ≤ 0.0001; ** P ≤ 0.01; * P ≤ 0.05; ns: non-significant; two-way ANOVA. (D) Head area quantification of mock (*n* = 27), ZIKV NS4A- (*n* = 29), ZIKV 2K-NS4B- (*n* = 25) and NS4A-NS4B- (*n* = 22) injected larvae at 3 days post-fertilization. *N* = 2. Data are shown as means ± SEM. *** P ≤ 0.001; ns: non-significant; one-way ANOVA. (E-F) TUNEL staining at 2 days post-fertilization (E) and cell death quantification (F) in the developing brain following ZIKV NS4A injection (*n* = 13) compared to H_2_O-injected (*n* = 13) zebrafish embryo. *N* = 2. A = anterior; P = posterior. Scale bars = 50 μm. * P ≤ 0.05. Student’s t-test. (G) At 1 dpf, 20 larvae injected with either water or NS4A-encoding RNA were pooled and subjected to protein extraction. Resulting samples were analyzed by western blotting using the indicated antibodies. Ponceau staining of total proteins on the membrane is shown as loading control. The arrow indicates the NS4A-specific signal. (H) Hatching rate at 3 days post-fertilization of larvae analyzed in (C). *n* indicates the number of fish; *N* represents the number of experimental repeats.

The viral determinants of ZIKV neurovirulence remain poorly understood. A previous study has shown that ubiquitous expression of ZIKV NS4A protein in drosophila induces severe neurodevelopmental defects including a reduced larval brain volume and apoptosis in neurons [26,27]. However, drosophila is an invertebrate model whose brain development substantially differs from that of humans. Therefore, we assessed neurovirulence potential of NS4A in the zebrafish larva, a vertebrate model closely resembling brain development in higher vertebrates and humans. We also included in the analysis mature NS4B alone since it interacts with NS4A and was shown to inhibit neurogenesis *in vitro* [11]. To confirm that *in vitro* transcribed RNAs can be correctly translated by the zebrafish cellular machinery, we microinjected a full-length *in vitro* transcribed ZIKV RNA genome (*i*.*e*., vRNA). vRNA injection fully recapitulated the morphological defects observed in infection (S6A–S6D Fig), demonstrating that this approach is in principle appropriate to investigate the impact of individual viral proteins expression. *In vitro* transcribed RNA encoding NS4A, NS4B (with the 2K signal peptide), or NS4A-(2K)-NS4B (NS4A precursor) were microinjected into 1 cell stage zebrafish embryos (Fig 6A). We then examined the effects of NS4A, NS4B, and NS4A-NS4B precursor expression on the general morphology. Embryos were analyzed for their morphology and imaged to qualitatively evaluate their motor activity. Strikingly, like for ZIKV-infected larvae, NS4A-injected animals displayed severe mobility restrictions (Video S3). NS4A expression caused morphological defects at 3 dpf such as curved or shortened tails as compared to the water injection control. Indeed, 60% of those embryos had mild or severe defects (Fig 6B and 6C). More importantly, NS4A expression led to 20% decrease of the head area (Fig 6D). This correlated with an increase in apoptosis in the brain at 2 dpf as measured using TUNEL assays (Fig 6E and 6F). As control, we confirmed that NS4A was expressed in injected animals as it was specifically detected at 1 dpi by western blotting (Fig 6G). In contrast, the injection of NS4B or NS4A-NS4B encoding RNAs did not lead to an increase in morphological defects compared to the water-injected larvae (Fig 6B–6D), demonstrating that the phenotypes are specific to mature ZIKV NS4A. It is noteworthy that hatching of embryos injected with NS4A was seriously affected, with 40% of NS4A-injected larvae in their chorion at 3 dpf, a time point by which hatching has normally already occurred (Fig 6H) [68]. Taken together, our results unequivocally demonstrate that NS4A is sufficient to trigger morphological and locomotor defects closely resembling those observed in ZIKV infection and to induce neurovirulence, constituting an attractive drug target for therapeutic development.

## Discussion

Despite multiple investigations using existing immunodeficient murine models and recently developed organoid culture models, the viral and host determinants driving ZIKV neuropathogenesis are still poorly understood. Our study highlights the zebrafish larva as a vertebrate model for ZIKV infection with neurological phenotypes comparable to those in humans. Taking advantage of transgenic lines with specifically labelled neural cell populations, combined with molecular and cellular analysis, we uncovered several host and viral determinants of ZIKV pathogenesis.

Upon injection in the yolk at 2 hours post-fertilization, ZIKV infects and replicates efficiently in the zebrafish embryos and larvae. This is demonstrated by several strong lines of evidence: 1- viral RNA, along with NS3 and E proteins, was detected in the larvae at 3 and 4 dpf (Figs 1F, 1H, 1I, 2A–2C and 4G). As a nonstructural protein, NS3 is absent from the virion and its detection confirms that translation from genomic viral RNA occurs in ZIKV-containing cells; 2- vRNA replication was confirmed by the presence of the NS5 polymerase-produced negative strand RNA at all tested time points (*i*.*e*., 1, 2 and 3 dpf; Fig 1G). This transient RNA species, a viral replication intermediate, is uniquely produced by the viral RNA polymerase NS5, which is also absent from the input virions and expressed only in infected cells; 3- Importantly, treatment with NITD008, a potent inhibitor of NS5 polymerase activity, reverted ZIKV-induced morphological and mobility defects, and decreased the viral load (Figs 1B–1E, 1H, 1I and 3B–3E). Moreover, ZIKV is detected in the developing brain, notably in NPCs, a known target of ZIKV in humans. Despite cells being pluripotent at the time of injection, ZIKV is primarily detected in NPCs at 4 days post-fertilization, indicating a specific cellular tropism. However, ZIVK infection was not restricted to neural progenitor cells since we detected a fraction of ZIKV envelope in Sox2-negative cells. These cells could be astrocytes, oligodendrocytes or microglial cells, which are also permissive to ZIKV [69], or infected NPCs that have initiated their differentiation into post-mitotic neurons and thus, lost *sox2* expression. Staining with specific markers of other neural population markers such as Neurod1, a marker for intermediate neurons or Elavl3, a marker of immature neurons, may be used in future studies to better discriminate ZIKV-infected cell populations. Future studies involving single-cell sequencing will allow a better understanding of ZIKV cellular tropism, and neuronal maturation, especially considering that previous studies reported that ZIKV impairs neuronal differentiation, consistent with our RNA sequencing data.

Injection of ZIKV at very early stage causes developmental defects in the zebrafish larvae characterized by morphological defects, mobility impairments, and more importantly, by a decrease in head area. Infection with DENV did not lead to any obvious abnormalities in the general development, except discrete pericardial edema in some scarce occurrences. No major induction of innate immunity responses was detected in live ZIKV H/PF/2013-infected animals. This strongly suggests that these phenotypes are specific to ZIKV infection and are not due to systemic inflammation or antiviral host responses. While we were performing this study, Maleski and colleagues reported a similar zebrafish larva-based ZIKV infection model [70]. As our observations here, they described that injection of a Brazilian ZIKV strain induced a reduction in the head size as well as major defects in motor activity. Strikingly, this correlated with marked increases in the expression of *ifnΦ1*, *ifnΦ3* and *TNFa* at the mRNA level, which we did not observe with our model (S2B–S2C Figs) although it possesses an inducible innate immune system as demonstrated in poly(I:C) stimulation experiments (S2G–S2H Figs). This discrepancy could be due to experimental differences between the two studies in terms of the zebrafish lines used for husbandry (AB *vs*. TL in our case) or the microinjected ZIKV pathogenic strains (a Brazilian strain *vs*. H/PF/2013 in our study) which may differ in *in vivo* replication fitness and induced cytopathic effects. S3 Fig shows that in our model, embryos injected with the Brazilian isolate HS-2015-BA-01 [38] (referred to as Brazil 2015) did not develop and all died before 48 hours post-fertilization, while the expression of the antiviral genes was induced 6 to 12-fold in stark contrast to the ZIKV H/PF/2013-related data. This is reminiscent to the poly(I:C) stimulation data (S2F–S2H Figs), which shows a higher expression of antiviral genes in dead animals. Considering this, it is tempting to speculate that when ZIKV strongly induces innate immunity in our system, the animals do not develop and die, and that only infected animals with no or low ISG/IFN expression can survive. Overall, these data support that the brain morphological defects reported here are due to ZIKV and not to a systemic immune response. Nevertheless, we cannot exclude a contribution of neuroinflammation since we might not detect local immune responses in the brain by RT-qPCR with RNA extracted from whole animals.

Treatment of ZIKV-infected animals with the NS5 polymerase inhibitor NITD008 systematically reverted the phenotypes. It unambiguously demonstrates that productive ZIKV vRNA replication is required to induce developmental defects. Such experiments additionally represent a proof-of-principle that zebrafish can be used for drug *in vivo* efficacy studies, enabling convenient and rapid testing of antiviral drug candidates before challenges in more elaborated, expensive, and time-consuming mammalian models. Moreover, considering that the drug was added to the water and efficiently taken-up by the larva, this model is ideal for *in vivo* medium throughput drug screening campaigns to identify novel antivirals, which would rely on ZIKV-induced defect reversion upon treatment. This infection model, combined with behavioral assays using the Daniovision recording chamber, enables quantitative and automated drug testing, highlighting the scalability of our approach. Follow-up studies with putative prioritized drug candidates should be then performed to better assess their suitability to inhibit ZIKV, taking advantage of various established murine models such as adult lethal infection models, vertical transmission models or *in utero* intracranial fetal infection models [3,37,71–73].

Most importantly, we show in this study that the zebrafish ZIKV infection model recapitulates important features of ZIKV neuropathogenesis and provides new insights about the associated dysfunctions. ZIKV replication in zebrafish led to increased cell death in the developing brain, which is consistent with previous reports in other animal models [6]. Together with the depletion of NPCs, these could explain the decrease in head area observed in infected larvae. It is noteworthy to mention that increased apoptosis in the brain and decreased NPC abundance were also observed in some ZIKV-infected animals without general morphological defects (Figs 3E, 4B, 4F and S5D, ZIKV “normal” animals). In humans, non-microcephalic infants with intrauterine exposure to ZIKV can exhibit neurodevelopmental delays such as cognitive and language impairments [74,75]. More studies of those infected fish could allow a better understanding of sequelae and long-term consequences of ZIKV infection. Our transcriptomic analysis of NPCs from infected larvae reveals that one of the most affected genes was *vglut1* with a decrease in mRNA levels of ~130-fold in our NPC transcriptome analysis. *vglut1* mRNA encodes the vesicular glutamate transporter type 1 and is a specific biochemical marker of excitatory glutamatergic neurons and glutamatergic synapses. Several evidences suggest an inhibitory/excitatory imbalance and particularly abnormalities in excitatory glutamatergic neurons and synapses in neurodevelopmental disorders including intellectual disabilities and epilepsy, which are sequelae of children born with the congenital Zika syndrome [76–79]. This imbalance correlates with severe mobility defects in both infected zebrafish and affected children. Interestingly, no modulation of genes involved in immune antiviral responses could be observed, suggesting that ZIKV-induced pathological sequelae are not driven by interferon signaling. It is thus plausible that the neurodevelopmental defects induced by ZIKV are partly due to a perturbation of the development, and functionality of the mature glutamatergic and/or GABAergic neuronal networks. This may pave the way towards novel therapeutic approaches aiming at dampening the symptoms in patients by chemically targeting one of these two mature neuron types, depending on the sequelae.

Here, we demonstrate that ZIKV NS4A is a major determinant of ZIKV neurovirulence in vertebrates as its expression alone at the early stages of development closely recapitulates the phenotypes observed upon infection. Although previous studies in drosophila have shown that ZIKV NS4A expression reduces the size of the larval brain [26,27], this is the first time to our knowledge that it is shown *in vivo* in a vertebrate model whose neuroanatomy and neurodevelopment resemble that of humans. As in infection, NS4A expression induced morphological defects which correlated with mobility impairment, a reduction in head size and the induction of apoptosis in the brain. In drosophila, NS4A induces microcephaly by inhibiting the host protein Ankle2 and its pathway [26,27]. Moreover, a recent study reported that Ankle2 knock-out in zebrafish resulted in microcephaly and a decrease in the number of radial glial progenitor cells, suggesting a role in neurogenesis in this model [80]. Since NS4A is also associated with Ankle2 human orthologue, it will be interesting to evaluate whether this interaction regulates NS4A neurovirulence in zebrafish larva. In human cells, flaviviral NS4A is strictly required for vRNA replication and accumulates in viral replication organelles which host the vRNA replication complexes [16,81–84]. Both mutagenesis and pharmacological approaches recently demonstrated that NS4A regulates the biogenesis of these replication factories [12,85]. Most notably, Riva and colleagues have shown that ZIKV NS4A function in replication organelle morphogenesis can be specifically inhibited by the compound SBI-0090799 [85]. Moreover, NS4A inhibits the Akt-mTOR pathway and the growth of neurospheres [11]. Finally, in the case of DENV, NS4A can dampen RIG-I-dependent interferon induction in cell culture suggesting that it contributes to neuropathogenesis by interfering with innate immunity *in vivo* although this remains to be addressed [86,87]. Overall, these multiple roles of NS4A highlight that this viral protein constitutes an attractive target for the development of direct-acting agents. Such NS4A drugs would provide a dual therapeutic benefit by inhibiting both its functions in viral replication and pathogenesis.

Since the animals infected with ZIKV HS-2015-BA-01 did not develop at all and died after 1 day post-fertilization, we could not use this strain to study the impact of ZIKV infection on brain development. Strikingly, the polyprotein sequences of H/PF/2013 and HS-2015-BA-01 (both belonging to the Asian lineage and epidemiology associated with the congenital Zika syndrome) are very close and differ by only 9 residues. They are specifically located at positions 151 in prM, 117 in NS2A, 120 in NS3 protease domain, 4 and 48 in NS4A, and 74 and 114 in the methyltransferase (MTase) domain of NS5, as well as 322 and 642 in its RNA-dependent RNA polymerase (RdRp) domain. Most relevant to this study, at position 4, NS4A has a phenylalanine (F4) in case of H/PF/2013 while HS-2015-BA-01 has a leucin instead (L4). Similarly, the nature of residue 48 also differs between the two strains. So, it is tempting to speculate that the amino terminus of NS4A contributes to ZIKV-induced neuropathogenesis and/or replication fitness. In our view, this is particularly interesting because this moiety containing the two residues is the only cytosolic domain of NS4A. This naturally disordered domain contributes to oligomerization and its conformation is influenced by its association with lipids [88–90]. Using reverse genetics and directed mutagenesis approaches, it will be relevant in future studies to evaluate the contribution of these natural polymorphisms of NS4A and more generally, its cytosolic domain to ZIKV neuropathogenesis.

While it is clear that NS4A plays a critical role in ZIKV-induced neurodevelopmental defects in zebrafish, one cannot exclude the possibility of other viral determinants synergizing NS4A activity to promote ZIKV neuropathogenesis. NS4A and NS4B cooperate and suppress the Akt-mTOR pathway which leads to inhibition of NPC growth *in vitro* and stimulation of autophagy [11]. However, in our system, the injection of RNAs encoding for either NS4B or NS4A-NS4B precursor did not induce any notable phenotypes. In contrast to NS4A, we were unfortunately unable to confirm that these viral proteins were properly expressed in the injected larvae, although we have previously shown that these IRES-driven RNAs were functional when cytoplasmically transcribed in T7 polymerase-expressing human cells [91]. Considering that in the case of DENV, NS4A and NS4B interact together [16], it will be relevant to test whether NS4B potentializes NS4A activity when co-expressed in zebrafish. Interestingly, one defect observed in ZIKV-injected larvae was body curvature, a typical phenotype of ciliopathy in zebrafish [92,93]. It is thus tempting to speculate that this phenotype is likely due to ZIKV NS5 protein, known for inducing ciliopathy, forcing premature neurogenesis in chicken embryo and affecting motile cilia located in the brain in human fetal microcephalic tissue [94]. This suggests that this viral protein also contributes to viral neuropathogenesis. Finally, there is a natural polymorphism at position 117 of NS2A (alanine for H/PF/2013 *vs*. valine for HS-2015-BA-01). Interestingly, V117 was shown to increase virulence *in vivo* in type I interferon-deficient mice without impeding viral replication in mammalian cell culture [95,96]. This is of relevance since the sole expression of NS2A in mouse fetuses induced a premature differentiation of the radial glial cells and aberrant positioning of neurons in fetal cortex [97]. Considering all this, future studies will be required to assess the relative contribution of NS4A to neuropathogenesis compared to other viral proteins.

In summary, our data unveil the zebrafish larva as a versatile model of ZIKV pathogenesis since it recapitulates ZIKV neurotropism and CNS developmental defects observed in humans. ZIKV induces apoptosis and NPC depletion, which are very likely to contribute to the development of microcephaly. Our study also provides important insights into host and ZIKV determinants of neuropathogenesis. Taking advantage of its flexibility for genetic manipulations, this innovative model provides an unprecedented access to the infected brain and “hard-to-catch” cell populations to study ZIKV pathogenesis as well as to perform *in vivo* drug screening and testing.

## Methods

### Ethics statement

All the experiments were performed in compliance with the guidelines of the Canadian Council for Animal Care and under the approval of institutionnal ethic committee (“Comité institutionnel de protection des animaux” (CIPA); certificate number: #2005–01) and biosafety committee (“Comité de biosécurité”; certificate number: #2016–12) of INRS.

### Fish husbandry

Adult zebrafish (*Danio rerio*) were reared at 28°C with a 12/12 light-dark cycle in the aquatic facility of the National Laboratory of Experimental Biology at Institut National de la Recherche Scientifique (INRS). Fertilized eggs were collected, treated as specified, kept in petri dishes, and raised at 28.5°C. The zebrafish lines used in this study were: wild-type TL, Tg(*nestin*:GFP), Tg(*gfap*:GFP), Tg (*flk1*:EGFP), Tg(*vglut2*:RFP) and Tg(*dlx5a/6a*:GFP;*vglut2*:RFP). The *nestin* and *gfap* transgenic lines, previously generated and described [48,49], were obtained from the laboratories of Pierre Drapeau and Eric Samarut. Tg(*dlx5a/6a*:GFP) was obtained from Dr. Marc Ekker.

### ZIKV and DENV production

Zika virus strain H/PF/2013 (Genbank accession KJ776791.2) was obtained from European Virus Archive Global (EVAg). The ZIKV Brazilian strain HS-2015-BA-01, referred to herein as Brazil 2015 (Genbank accession KX520666.1), was kindly provided by Dr. Anne Gatignol (McGill University) and Dr. Mauro Teixeira (Universidade Federal de Minas Gerais) [38]. Virus stocks were passaged in Vero E6 cells [98]. The plasmid coding for serotype 2 DENV2 16681s genome sequence (pFK-DENV-WT) was previously described [99]. Following pFK-DENV linearization with XbaI, *in vitro* transcription was performed using the mMessage mMachine kit (Thermo-Fisher) with SP6 RNA polymerase. DENV2 16681s stock were produced after electroporation of Vero E6 cells with *in vitro* transcribed genomes as described in [100]. ZIKV and DENV viral titers were determined by plaque assays. Briefly, 24-well plates were seeded with 2 x 10^5^ Vero E6 cells/well. Cells were infected with serially diluted virus samples. Two hours post-infection, inoculums were replaced for MEM (Invitrogen) containing 1.5% carboxymethylcellulose (Millipore-Sigma). 4 days (ZIKV) and 7 days (DENV) after infection, cells were fixed with 10% formaldehyde and plaques were counted following staining with 1% crystal violet/10% ethanol.

### *In vitro* ZIKV proliferation assay and cytotoxicity

To compare H/PF/2013 ZIKV and HS-2015-BA-01 ZIKV replication kinetic, 2 x 10^5^ Huh7.5 human hepatocarcinoma cells were seeded in 6-well plate and cultured overnight. The following day, they were infected with H/PF/2013 or HS-2015-BA-01 (MOI = 0.1). Virus inoculum was removed 2 hours later. Supernatants containing viruses were harvested from 1 to 3 days post-infection. Infectious virus titers were determined by plaque assays as describe above.

Cell viability following ZIKV infection was evaluated using MTT assays. Briefly, 5 x 10^4^ Huh7.5 were seeded in 24-well plate, cultured overnight, and infected with H/PF/2013 or HS-2015-BA-01 (MOI = 0.1), or left uninfected (three wells per condition per experiment). At 1, 2 and 3 days post-infection, 100μL of 3-(4,5-dimethylthiazol-2-yl)-2,5-diphenyltetrazolium bromide (MTT) at 5 mg/mL was added in the medium for 2 hours at 37°C. Medium was then removed and 750 μL of 2% (v/v) glycine (0.1M, pH 11) in DMSO was added to dissolve the MTT precipitates. Absorbance at 570 nm was read with a Spark multi-mode microplate reader (Tecan) with the reference at 650 nm. Values for each time point were normalized to that for the corresponding uninfected control.

### Embryo infection

Using the FemtoJet 4i microinjector (Eppendorf), a volume of 2 nL containing ZIKV particles (~5 x 10^7^ PFU/mL), DENV particles (~4 x 10^6^ PFU/mL) or DMEM (vehicle) was injected into 2-to-4 cell stage embryos. Four hours post-injection, fish were either treated with 100 μM NITD008 (Tocris Small Molecules) or 0.5% DMSO as control.

### Viral protein expression in embryos

The plasmids containing NS4A, 2K-NS4B, or NS4A-NS4B sequences downstream T7 RNA polymerase promoter were previously described [91]. Following pTM-ZIKV NS4A, pTM-ZIKV 2K-NS4B, and pTM-ZIKV NS4A-NS4B plasmids linearization with SpeI, *in vitro* transcription was performed using the TranscriptAid T7 High Yield transcription kit (Thermo Scientific). RNA translation initiation is driven by the encephalomyocarditis virus (ECMV) internal ribosome entry site (IRES) at the 5’ terminus. A volume of 1 nL containing 500 ng/μL mRNA coding for each of these proteins was injected into the cell of one-cell stage embryos using the FemtoJet 4i microinjector (Eppendorf). H_2_O was used as reference control.

For western blot experiments, 20 larvae per condition were collected and pooled at one day post-injection (*i*.*e*., 1 dpf). Following resuspension in 100 μL of RIPA lysis buffer (150 mM NaCl, 50 mM Tris (pH 7.4), 0.5% sodium deoxycholate, 0.1% SDS, 1% NP40 and EDTA-free protease inhibitors (Roche)), larvae were immediately grinded three times for 20 seconds on ice with a tissue tearor (Biospecs Products Inc., model 965370) and subsequently sonicated four times for 15 seconds on ice. The resulting lysates were centrifuged at 13,000 rpm for 20 minutes at 4°C. The supernatants were collected, and protein concentration was determined using Bradford assay (Bio-Rad). Twenty μg of lysate per condition were resolved on a 12% SDS-polyacrylamide gel. After electrophoresis, proteins were electrotransferred onto a PVDF membrane which was stained with Ponceau solution to visualize total transferred proteins. NS4A was detected using rabbit polyclonal anti-ZIKV NS4A antibodies (Genetex, #GTX133704). Following incubations with HRP-coupled anti-rabbit secondary antibodies (Millipore-Sigma) and the Clarity Max ECL Substrate reagent (Bio-Rad), NS4A signal was imaged with a ChemiDoc (Bio-Rad).

### Survival and morphology

Injected embryos were monitored for their survival rate for 3 days post-fertilization using heartbeat as a readout of viability. At the latest time point (*i*.*e*., 3 days post injection larval stage), larvae were fixed in 4% paraformaldehyde (PFA; in PBS) overnight at 4°C and observed under Stemi 305 microscope (Zeiss) to assess hatching and general morphology according to the criteria shown in S1A Fig. For head area measurement, larvae were positioned laterally, and head sizes were calculated using the Fiji software.

### Behavioral analysis

At 4 days post-fertilization, larvae were separated into single wells of a 96-well plate containing 200 μL of E3 media. Following 30 minutes of habituation in the DanioVision recording chamber (Noldus), locomotor activity upon light stimulation was monitored for two hours. Swimming distance (in mm) and immobility times (in seconds) analysis were performed using the EthoVision XT12 software (Noldus). Swimming and responses-to-touch videos were taken with an iPhone (Apple) coupled to a stereomicroscope at 30 frames per second.

### RT-qPCR and RT-ddPCR

At the indicated time points, RNAs were isolated from pools of equal number of whole infected or non-infected larvae (typically 6 to 15) using the RNeasy mini kit (Qiagen) according to the manufacturer’s protocol. 200–500 ng of extracted RNA were subjected to reverse transcription using the Invitrogen SuperScript IV VILO Master Mix RT kit (Life Technologies) or QuantiTect Reverse Transcription kit (Qiagen). ZIKV cDNA, DENV cDNA and *vglut1* cDNA were detected by droplet digital PCR (ddPCR) and absolute quantification was performed using the QX200 ddPCR Evagreen Supermix (Bio-Rad) and the QX200 ddPCR System (Bio-Rad). The following primer pairs were used: 5′-AGATGAACTGATGGCCGGGC-3′ and 5′AGGTCCCTTCTGTGGAAATA-3′ for ZIKV H/PF/2013; 5’-CAGGTTATGGCACTGTCACAAT-3’ and 5’-CCATCTGCAGCAACACCATCTC-3’ for DENV2 16681s; 5’-CTTTAAAGCCCAGGCAGGGA-3’ and 5’-AGGATGGCGATGATGTAGCG-3’ for *vglut1*. Absolute quantification of ZIKV H/PF/2013 negative-strand RNA (ZIKV (-) RNA) was determined by RT-ddPCR. Total RNA was subjected to reverse transcription using the QuantiTect Reverse Transcription kit (Qiagen) with negative strand-specific tagged primer 5’-ggccgtcatggtggcgaataaAGATGAACTGATTGGCCGGGC-3’ allowing the incorporation of a unique tag sequence into the cDNA synthesized from (-)RNA (lowercase letters in the primer sequence) [40,101]. ZIKV negative-strand cDNA was amplified and detected by ddPCR using with the tag-specific primer 5’-GGCCGTCATGGTGGCGAATAA-3’ and ZIKV reverse primer 5’-AGGTCCCTTCTGTGGAAATA-3’. The assay was optimized with RNA from cultured infected Huh7.5 cells. The amplicon size (144 base pairs) and sequence were confirmed using agarose gel electrophoresis and Sanger sequencing, respectively. For other cDNA quantifications, real-time PCR was performed using the Applied Biosystems SYBR Green Master mix (Life Technologies) and a LightCycler 96 (Roche) for the detection. 5’-GCCCAAGTAAACACCCTGGA-3’ and 5’-GCAAAGCTCTGTATGTTGCCA-3’ for *nestin*; 5’-AATGTCAAACTGGCCCTGGA-3’ and 5’-TCTGCACCGGAACAGTGATT-3’ for *gfap*; 5’-TGAGAACTCAAATGTGGACCT-3’ and 5’-GTCCTCCACCTTTGACTTGT-3’ for *ifnφ1*; 5’-GAGGATCAGGTTACTGGTGT-3’ and 5’-GTTCATGATGCATGTGCTGTA-3’ for *ifnφ3;* 5’-CGGACCTCAGTTTCAAGG-3’ and 5’-GCAGCGGGAGAATATGGA-3’ for *rig-I*; 5’-GCAGCATGGTGAGATACGAA-3’ and 5’-GTTGGAATGCCTGATCCACA-3’ for *TNFa;* 5’-GGAGAATCAGTTACAAAACCT-3’ and 5’-GATTGTCTCTTGCCTTGTAACA-3’ for *mx1*; 5’-GCTGAAAGAAGCAGGAATGG-3’ and 5’-AAACACTGGAAGACCTTCCAA-3’ for *viperin (rsad2)*; 5’-AACTCGGTGACGATGCAGC-3’ and 5’-TGGGCACGTTGAAGTACTGA-3’ for *isg15;* 5’-GTGGCTGGAGACAGCAAGA-3*’* and 5’-AGAGATCTGACCAGGGTGGTT-3’ for *ef1a*. The ΔΔCt method was used to determine the relative expression levels normalized to *ef1a*, used as a housekeeping gene [24,102,103].

### Wholemount immunostaining

Larval zebrafish (3 dpf) were fixed in 4% PFA/PBS overnight at 4°C. After fixation, the larvae were washed several times for 1 hour with PBS-0.1% Tween20 (PBST) and then incubated in PBS containing 1 mg/ml collagenase (75 minutes) to remove the skin. The collagenase was washed off with PBST for 1 hour, and the larvae were incubated in blocking solution (2% normal goat serum, 1% bovine serum albumin, 1% DMSO, 1% Triton-X in PBS) for 30 minutes. The larvae were rinsed several times for 30 minutes with PBST and then incubated in blocking solution containing rat anti-DENV NS3 antibodies (which are cross-reactive with ZIKV NS3 as previously described [91]) overnight at 4°C. Subsequently, larvae were incubated in blocking solution containing secondary antibodies (Alexa fluor 488 anti-rat antibodies, Life Technologies, #A21208) overnight at 4°C. The following day, the larvae were washed several times with PBST, deyolked with tweezers, and mounted on slides in glycerol. Z-stack images were taken using a LSM780 confocal microscope (Carl Zeiss Microimaging) at the Confocal Microscopy and Flow Cytometry Core Facility of INRS. Images were acquired using the same settings and processed using the Fiji (ImageJ) software.

### TUNEL assays

To analyze cell death, TUNEL assays were performed on whole embryos at 2 days post-fertilization. At one day post-fertilization, embryos were treated with 0.003% phenyltheioura (PTU) to block pigment formation. One day later (*i*.*e*., two days post injection), larvae were fixed overnight with 4% paraformaldehyde (PFA, in PBS), dehydrated, and rehydrated using serial dilutions of methanol in PBST then washed with PBST. Following inactivation of endogenous nucleases with proteinase K at 10 μg/mL for 20 minutes, whole larvae were washed with PBST and PBS, fixed with 4% PFA/PBS for 20 minutes, and incubated with the *In Situ* Cell Death Detection Kit Fluorescein mix (Roche) at 37°C for 1 hour. Larvae were imaged with a LSM780 confocal microscope (Carl Zeiss Microimaging) at the Confocal Microscopy and Flow Cytometry Core Facility of INRS. Counting of TUNEL-positive cells was performed manually using the Cell Counter plug-in for Fiji/Image J (NIH) Imaging software.

### Brain cryo-sections and immunofluorescence

At 2 days and 4 days post-fertilization, zebrafish were fixed in 4% PFA/PBS, dehydrated in serial dilutions of sucrose, and frozen in Tissue plus O.C.T compound (Fisher Scientific). 12 micron-thick transverse sections of the head were prepared, dried at room temperature, and frozen at -80°C until use.

For Sox2 staining and Sox2/ZIKV E co-staining, sections were washed with PBS. Antigen retrieval was performed solely on Sox2 single staining by incubation with Tris-HCl (pH 8.2, 50mM) at 85°C for 6 minutes. Sections were then washed with PBS-0.5% TritonX-100, incubated in blocking solution (10% normal goat serum in PBS) for one hour, and in primary antibody solution anti-Sox2 (1:200, Invitrogen), panflaviviral anti- E (clone 4G2; 1:400, Genetex) diluted in 5% normal goat serum, 1% bovine serum albumin in PBS containing 0.1%TritonX-100 overnight at 4°C. After several washes with PBS-0.3% TritonX-100, sections were incubated with species-specific Alexa Fluor-conjugated secondary antibodies (goat anti-rabbit IgG Alexa Fluor 568 (#A21236), or goat anti-mouse IgG Alexa Fluor 647 (#A21236) highly cross-adsorbed secondary antibodies; Life Technologies) for 2 hours at room temperature in the dark. Sections were washed for one hour with PBS-0.3% TritonX-100, incubated for 10 minutes in 1:1,000 Hoescht to stain the nuclei, and mounted.

Transversal brain sections were imaged with a LSM780 confocal microscope (Carl Zeiss Microimaging) and were analyzed using Zeiss Zen (black edition) and Fiji software. Schematic brain representation and structures’ identification were made based on the Atlas of Early Zebrafish Brain Development by Mueller and Wulliman [55].

### Embryos dissociation and flow cytometry

Tg(*gfap*:GFP), Tg(*nestin*:GFP) and Tg (*flk1*:EGFP) embryos dissociation was performed as described before [51] with minor modifications. One day post-fertilization embryos were dechorionated, and deyolked in deyolking buffer (55 mM NaCl, 1.8 mM KCl, 1.25 mM NaHCO_3_) in the presence of 10,000 123count eBeads counting beads (Thermo-Fisher). Deyolked embryos were pelleted at 500g for 5 minutes and rinsed with FACSmax cell dissociation solution (Genlantis). Embryos were transferred in 6-well plates and dissociated with FACSmax by gentle up-and-downs. Single cells suspensions were pelleted and washed with PBS. For flow cytometry experiments, cells were fixed in 4% PFA/PBS and filtered using a cell strainer (Fisher Scientific) before data acquisition using a LSRFortessa Cell Analyzer (BD) at the Confocal Microscopy and Flow Cytometry Core Facility of INRS. Data analysis was performed using the FlowJo software (version 10.8.1). Gating based on forward *vs*. side scatter (FSC *vs*. SSC) and forward scatter height *vs*. forward scatter area (FSC-H *vs*. FSC-A) was applied to exclude cell debris and doublets, respectively. Age-matched non transgenic (*i*.*e*., GFP-negative) embryos (same genetic background) were used to set fluorescence thresholds and consider cell which expressed high intensity of GFP emission (>10^4^ fluorescence units).

### Cell sorting and RNA sequencing

One day post mock- or ZIKV-injection, Tg(*nestin*:GFP) embryos were dissociated into single-cells suspension as described above. Cell sorting was performed on unfixed cells using a FACSMelody Cell Sorter (BD Biosciences) in a biosafety level 2 cabinet located at the Confocal Microscopy and Flow Cytometry Core Facility of INRS. GFP expressing cells were directly sorted into buffer RLT (Qiagen) containing 1% β-mercaptoethanol. RNAs were extracted using the RNeasy Mini kit (Qiagen) according to the manufacturer’s instructions. RNA quality control and next generation RNA sequencing were performed at the Genomics Core Facility of the Institute for Research in Immunology and Cancer (IRIC, University of Montreal) using 2100 bioanalyzer (Agilent) and NextSeq 500 instrument (Illumina), respectively. RNA sequencing data analysis was performed by the Bioinformatics Core Facility of IRIC.

### GABAergic and glutamatergic neurons quantification

To image GABAergic and glutamatergic neurons, the Tg(*dlx5a/6a*:GFP; *vglut2*:RFP) line was used. In this line, GFP is expressed under the control of *dlx5a/6a* promoter, whose activity is specific to GABAergic neurons. RFP is expressed under the control of *vglut2* promoter, active in glutamatergic neurons. ZIKV- or mock-infected 3dpf zebrafish larvae were fixed in 4% PFA overnight at 4°C. PBS-washed zebrafish larvae were ventrally mounted for brain imaging. Z-stack images were taken using a Zeiss LSM780 confocal microscope (Zeiss).

For GABAergic neurons (*dlx5a/6a*:GFP+) quantification, GFP^+^ cells were counted in the brain regions manually using the Cell Counter plug-in for Fiji/Image J (NIH) Imaging software.

Quantification of glutamatergic neuron abundance (v*glut2*:RFP+ cells) was performed using flow cytometry. Zebrafish larvae at 3dpf were dissociated as described above. Unfixed cells and 123count eBeads counting beads (Thermo-Fisher) were processed for RFP^+^ cell counting using BD FACSMelody Cell Sorter at the Confocal Microscopy and Flow Cytometry Core Facility of INRS. Data analysis was performed using the FlowJo software (version 10.8.1). Gating and thresholding were performed as described above.

### Statistical analyses

Data were analyzed using GraphPad Prism 8 software. The number of experiments (*N*) and sample size (*n*) are mentioned in the legends. Data are presented as mean ± SEM or median ± 95% confidence interval (CI) as specified in legends. Normality test was determined using the D’Agostino & Pearson test. Significance was determined using either unpaired Student’s t-test, one-way ANOVA, two-way ANOVA (for data presenting a normal distribution), nonparametric Mann-Whitney or nonparametric Kruskal-Wallis (for data presenting a non-normal distribution) tests as specified in legends.

## Supporting information

S1 Fig Morphological phenotype classification and mobility defects following ZIKV infection in zebrafish embryos. (A) Overview of several defects observed in larvae at 3 dpf and used as criteria for the classification in the subsequent morphological analyses of the study. Scale bar = 1 mm. (B) Representative images of the head of mock- and ZIKV-infected larvae at 3 dpf (lateral view). The dashed line indicates the head area that was measured in Fig 1E. Scale bar = 0.15 mm. (C-D) The distance moved (C) by mock-, ZIKV-, DENV-infected larvae and the immobility time (D) were assessed using the DanioVision device (mock; *n* = 27; ZIKV, *n* = 35; DENV, *n* = 34. *N* = 2). Data are shown as median ± 95 CI. **** P ≤ 0.0001; *** P ≤ 0.001; ** P ≤ 0.01; Kruskal-Wallis test. *n* indicates the number of fish; *N* represents the number of experimental repeats.
(TIF)

S2 Fig DENV replication kinetics and antiviral responses following ZIKV, DENV or poly(I:C) injection. (A) Cell medium (mock) or 10 PFUs DENV 16681s viral particles were microinjected in the zebrafish yolk at 2 hours post-fertilization (hpf). DENV RNA levels in whole larvae pools (6–15 larvae) at 1, 2 and 3 dpf were determined using ddPCR. Absolute DENV RNA copy numbers per embryo per day post-fertilization are shown. *N* = 3. Data are means ± SEM. ** P ≤ 0.01; * P ≤ 0.05; Student’s t-test for each day. (B-C) Cell medium (mock) or ZIKV H/PF/2013 were microinjected in the zebrafish yolk at 2 hours post-fertilization (hpf). Total RNA from 1 dpf (B) and 3 dpf pools (C) of 10 larvae was extracted and subjected to RT-qPCR to quantify the levels of *ifnφ1*, *ifnφ3*, *TNFa*, *mx*, *viperin*, *isg15 and rig-I* mRNAs (1dpf, *N* = 4; 3dpf, *N* = 7). Data are mean normalized values (relative to the uninfected conditions) ± SEM. ** P ≤ 0.01; ns: not significant; Student’s t-test. (D-E) Larvae were infected exactly as in (A). The expression of the same panel of genes as in (B-C) was analyzed at 1 dpf (D) and 3 dpf (E) (1dpf, *N* = 3; 3dpf, *N* = 3). Data are mean normalized values (relative to the uninfected conditions) ± SEM. ns: not significant; Student’s t-test. (F-H) At 2 hours post-fertilization embryos were microinjected with 2nL (1μg/μL) poly(I:C) as described in [104] or with 0.9% sodium chloride solution (vehicle). At 1 dpf, viability was visually assessed (F) and RNA from live (G) and dead (H) embryos was extracted and subjected to RT-qPCR as in (B-E). (Alive, *n* = 58; Dead, *n* = 107. *N* = 7) Data are mean normalized values (relative to the uninfected conditions) ± SEM. *** P ≤ 0.001; ** P ≤ 0.01; *: P ≤ 0.05. Student’s t-test. *n* indicates the number of fish; *N* represents the number of independent experimental repeats.
(TIF)

S3 Fig Infection with ZIKV HS-2015-BA-01 Brazilian strain is associated with a high mortality *in vitro* and in zebrafish embryos. (A-B) Human hepatocarcinoma Huh7.5 cells were infected with either ZIKV H/PF/2013 or ZIKV HS-2015-BA-01 (Brazil 2015) at a multiplicity of infection (MOI) of 0.1. 24, 48 and 72 hours later, cell viability compared to uninfected cells (A) and infectious viral production (B) were evaluated using MTT assays and plaque assays, respectively. Data are means ± SEM. *** P ≤ 0.001; ** P ≤ 0.01; *: P ≤ 0.05. Student’s t-test. (C-D) Twenty ZIKV viral particles (H/PF/2013 or Brazil 2015) were microinjected in the zebrafish yolk at 2 hours post-fertilization. (C) Representative pictures of mock-infected and Brazilian ZIKV strain (Brazil 2015)-infected larvae at 1dpf. (D) Survival curve over 3 days post-fertilization (dpf) of mock-infected (*n* = 85), ZIKV H/PF/2013-infected (*n* = 66) and ZIKV Brazil 2015-infected (*n* = 21) larvae (*N* = 2). (E) The production of ZIKV Brazil 2015 viral particles at 1 day post-infection was measured by plaque assays. (F) At 1 day post-fertilization, amounts of *ifnφ3*, *TNFa*, *mx*, *isg15*, *and viperin* mRNAs in mock and ZIKV Brazil 2015-infected larvae were analyzed by RT-qPCR.
(TIF)

S4 Fig ZIKV infection of Tg(*gfap*:GFP) and Tg(*nestin*:GFP) induces morphological defects and a depletion of NPCs. (A) Proportion of Tg(*gfap*:GFP) larvae with different morphological phenotypes at 3 days post-fertilization following ZIKV-injection (*n* = 27) or mock-injection (*n* = 32). *N* = 1. (B) Proportion of Tg(*nestin*:GFP) larvae with different morphological phenotypes at 3 days post-fertilization following ZIKV-injection (*n* = 85) or mock-injection (*n* = 87). *N* = 2. (C) At 1 day post-fertilization, ZIKV infected or uninfected whole transgenic Tg(*nestin*:GFP) embryos were dissociated in the presence of 10,000 fluorescent normalizing beads. Single cells and beads were counted by flow cytometry. The relative abundance of GFP^+^ cells counted per 100 beads are shown. (D-E) mRNA amounts of *nestin* and *gfap* in GFP^+^ (NPCs) and GFP^-^ in mock and ZIKV-infected Tg(*gfap*:GFP) (D) and Tg(*nestin*:GFP) embryos (E) were analyzed at 1 dpf by RT-qPCR. (F) Within the two first hours following fertilization, Tg(*flk1*:EGFP) embryos were injected with ZIKV (*n* = 101) or vehicle (mock, *n* = 123). At 3 dpf, the morphology of the larvae was analyzed as in Fig 1. (G) At 1 day post-fertilization, ZIKV infected or uninfected whole transgenic Tg(*flk1*:EGFP) embryos were dissociated in the presence of 10,000 fluorescent normalizing beads. EGFP^+^ cells (*i*.*e*. endothelial cells) and beads were counted by flow cytometry. Data are shown as means ± SEM. (Mock, *n* = 95; ZIKV, *n* = 95; *N* = 4). ns: not significant; Student’s t-test. *n* indicates the number of fish; *N* represents the number of independent experimental repeats.
(TIF)

S5 Fig ZIKV infection results in a loss of NPCs in larvae at 4 days post-fertilization. (A) Schematic representation of a zebrafish brain at 4 days post-fertilization. The three areas of the brain are shown: forebrain, midbrain and hindbrain. Gray lines represent the localization of the transverse sections. D = dorsal; V = ventral; A = anterior; P = posterior. (B-D) Number of neural progenitor cells (Sox2^+^ cells) in the midbrain (B) and the hindbrain (C) of 4 dpf mock-injected or ZIKV-injected fish. TeO = tectum opticum; T = midbrain tegmentum; N = region of the nucleus of medial longitudinal fascicle; MO = medulla oblongata. Scale bars = 50 μm. (D) Quantification of (B-C). Data are presented as means ± SEM. ** P ≤ 0.01; * P ≤ 0.05; ns: non-significant. Two-way ANOVA.
(TIF)

S6 Fig Injection of the ZIKV RNA genome into embryos induces morphological defects. (A) *In vitro* transcribed ZIKV RNA genome (vRNA) was microinjected in the zebrafish embryo at 1 hour post-fertilization. Created with BioRender.com. (B) Representative pictures of microinjected larvae at 3 days post-fertilization. ZIKV vRNA injection induced both severe and mild developmental phenotypes. (C and D) Quantification of the proportion of larvae with the different phenotypes (C), and head size (D) at 3 dpf of the larvae (Mock, *n* = 19; ZIKV RNA, *n* = 22. *N* = 2). Data are means ± SEM. * P ≤ 0.05; Student’s t-test. *n* indicates the number of fish; *N* represents the number of experimental repeats.
(TIF)

S1 Video Locomotor activity of control (mock), ZIKV-infected (ZIKV), and ZIKV-infected and 100 μM NITD008-treated (ZIKV+NITD008) embryos at 2 days post-fertilization. (MP4)

S2 Video Locomotor activity of control (mock), ZIKV-infected (ZIKV), and ZIKV-infected and 100 μM NITD008-treated (ZIKV+NITD008) larvae at 3 days post-fertilization. (MP4)

S3 Video Locomotor activity of water control (mock) and ZIKV NS4A-encoding RNA-injected larvae at 3 days post-fertilization. (MP4)

S1 Table RNA-seq differential expression analysis of NPCs isolated from ZIKV-infected and control embryos. (XLSX)

S1 Data Source data for Figs 1D–1I, 3C, 3E, 4B, 4F, 5A-5D, 5G, 5I, 6C, 6D, 6F, 6H, S1C, S1 Table, and S1D, S2A–S2H, S3A, S3B, S3E, S3F, S4A–S4G, S5D, S6C, S6D Figs. (XLSX)
