# Supplementary figures and images for: A zebrafish-based in vivo model of Zika virus infection unveils alterations of the glutamatergic neuronal development and NS4A as a key viral determinant of neuropathogenesis
Source: PLoS Pathog. 2024 Dec 2;20(12):e1012756. doi: 10.1371/journal.ppat.1012756 (PMC11637437; doi:10.1371/journal.ppat.1012756)

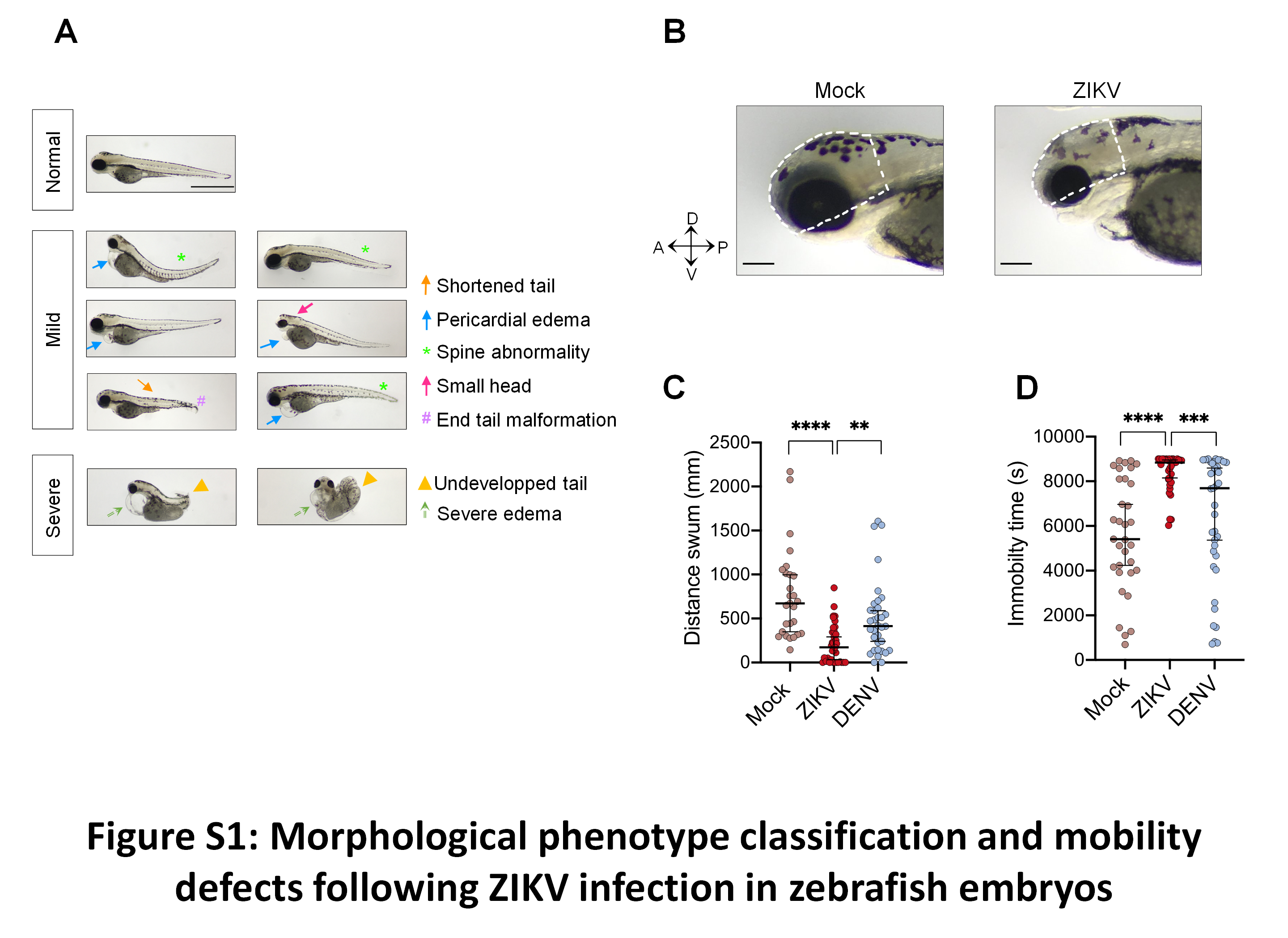

Supplement: S1 Fig — (A) Overview of several defects observed in larvae at 3 dpf and used as criteria for the classification in the subsequent morphological analyses of the study. Scale bar = 1 mm. (B) Representative images of the head of mock- and ZIKV-infected larvae at 3 dpf (lateral view). The dashed line indicates the head area that was measured in Fig 1E. Scale bar = 0.15 mm. (C-D) The distance moved (C) by mock-, ZIKV-, DENV-infected larvae and the immobility time (D) were assessed using the DanioVision device (mock; n = 27; ZIKV, n = 35; DENV, n = 34. N = 2). Data are shown as median ± 95 CI. **** P ≤ 0.0001; *** P ≤ 0.001; ** P ≤ 0.01; Kruskal-Wallis test. n indicates the number of fish; N represents the number of experimental repeats. (TIF) [file ppat.1012756.s001.tif]

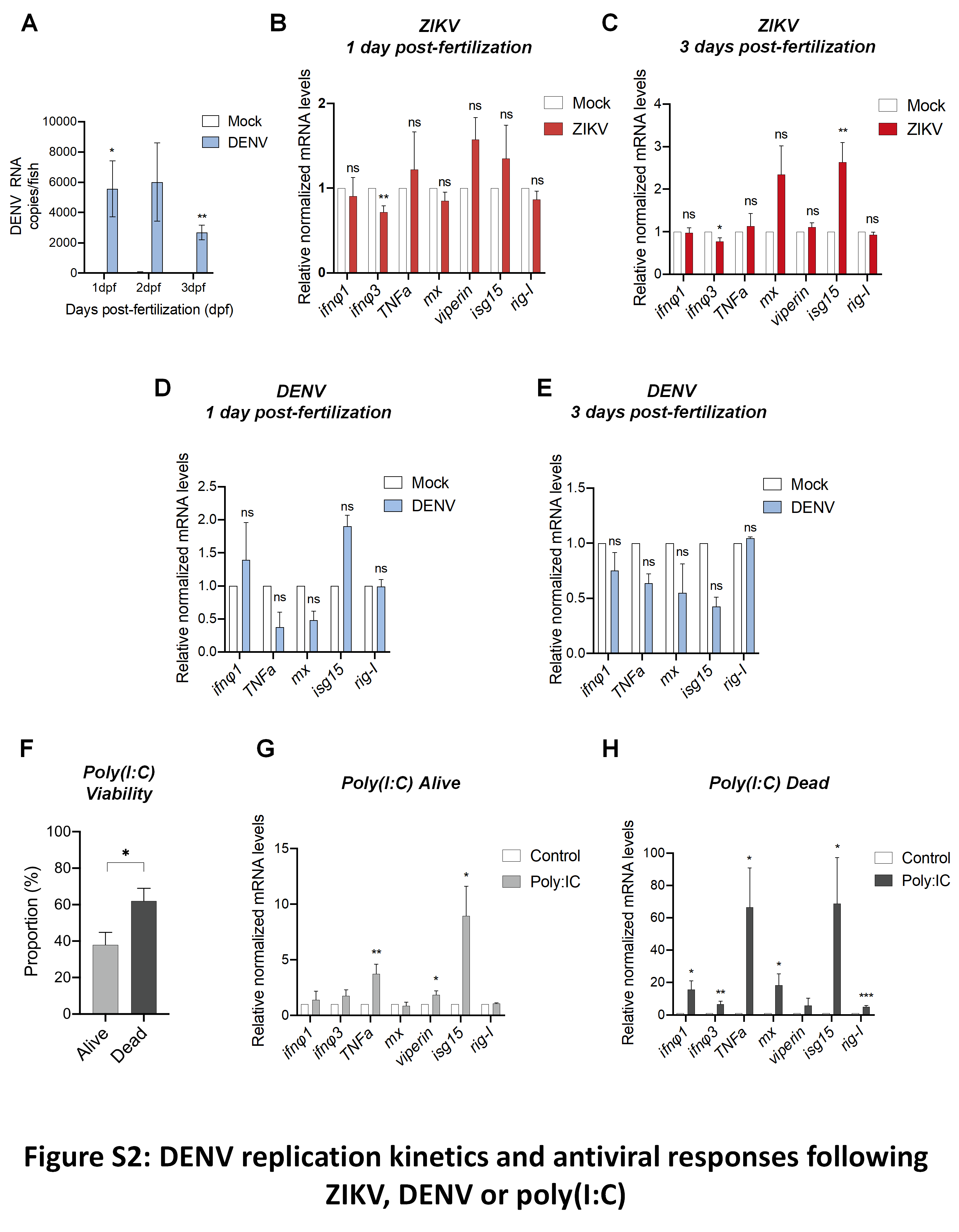

Supplement: S2 Fig — (A) Cell medium (mock) or 10 PFUs DENV 16681s viral particles were microinjected in the zebrafish yolk at 2 hours post-fertilization (hpf). DENV RNA levels in whole larvae pools (6–15 larvae) at 1, 2 and 3 dpf were determined using ddPCR. Absolute DENV RNA copy numbers per embryo per day post-fertilization are shown. N = 3. Data are means ± SEM. ** P ≤ 0.01; * P ≤ 0.05; Student’s t-test for each day. (B-C) Cell medium (mock) or ZIKV H/PF/2013 were microinjected in the zebrafish yolk at 2 hours post-fertilization (hpf). Total RNA from 1 dpf (B) and 3 dpf pools (C) of 10 larvae was extracted and subjected to RT-qPCR to quantify the levels of ifnφ1, ifnφ3, TNFa, mx, viperin, isg15 and rig-I mRNAs (1dpf, N = 4; 3dpf, N = 7). Data are mean normalized values (relative to the uninfected conditions) ± SEM. ** P ≤ 0.01; ns: not significant; Student’s t-test. (D-E) Larvae were infected exactly as in (A). The expression of the same panel of genes as in (B-C) was analyzed at 1 dpf (D) and 3 dpf (E) (1dpf, N = 3; 3dpf, N = 3). Data are mean normalized values (relative to the uninfected conditions) ± SEM. ns: not significant; Student’s t-test. (F-H) At 2 hours post-fertilization embryos were microinjected with 2nL (1μg/μL) poly(I:C) as described in [104] or with 0.9% sodium chloride solution (vehicle). At 1 dpf, viability was visually assessed (F) and RNA from live (G) and dead (H) embryos was extracted and subjected to RT-qPCR as in (B-E). (Alive, n = 58; Dead, n = 107. N = 7) Data are mean normalized values (relative to the uninfected conditions) ± SEM. *** P ≤ 0.001; ** P ≤ 0.01; *: P ≤ 0.05. Student’s t-test. n indicates the number of fish; N represents the number of independent experimental repeats. (TIF) [file ppat.1012756.s002.tif]

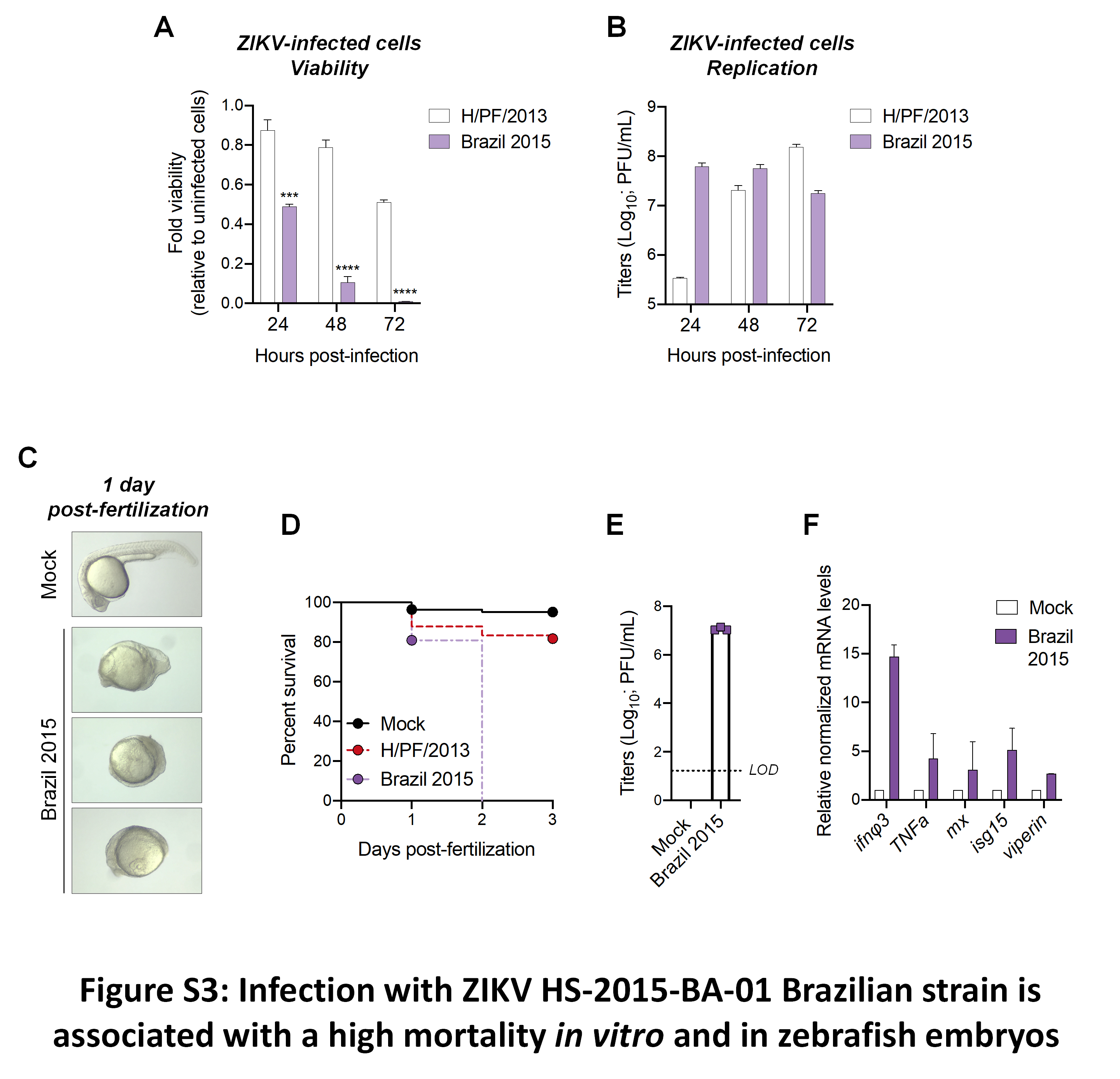

Supplement: S3 Fig — (A-B) Human hepatocarcinoma Huh7.5 cells were infected with either ZIKV H/PF/2013 or ZIKV HS-2015-BA-01 (Brazil 2015) at a multiplicity of infection (MOI) of 0.1. 24, 48 and 72 hours later, cell viability compared to uninfected cells (A) and infectious viral production (B) were evaluated using MTT assays and plaque assays, respectively. Data are means ± SEM. *** P ≤ 0.001; ** P ≤ 0.01; *: P ≤ 0.05. Student’s t-test. (C-D) Twenty ZIKV viral particles (H/PF/2013 or Brazil 2015) were microinjected in the zebrafish yolk at 2 hours post-fertilization. (C) Representative pictures of mock-infected and Brazilian ZIKV strain (Brazil 2015)-infected larvae at 1dpf. (D) Survival curve over 3 days post-fertilization (dpf) of mock-infected (n = 85), ZIKV H/PF/2013-infected (n = 66) and ZIKV Brazil 2015-infected (n = 21) larvae (N = 2). (E) The production of ZIKV Brazil 2015 viral particles at 1 day post-infection was measured by plaque assays. (F) At 1 day post-fertilization, amounts of ifnφ3, TNFa, mx, isg15, and viperin mRNAs in mock and ZIKV Brazil 2015-infected larvae were analyzed by RT-qPCR. (TIF) [file ppat.1012756.s003.tif]

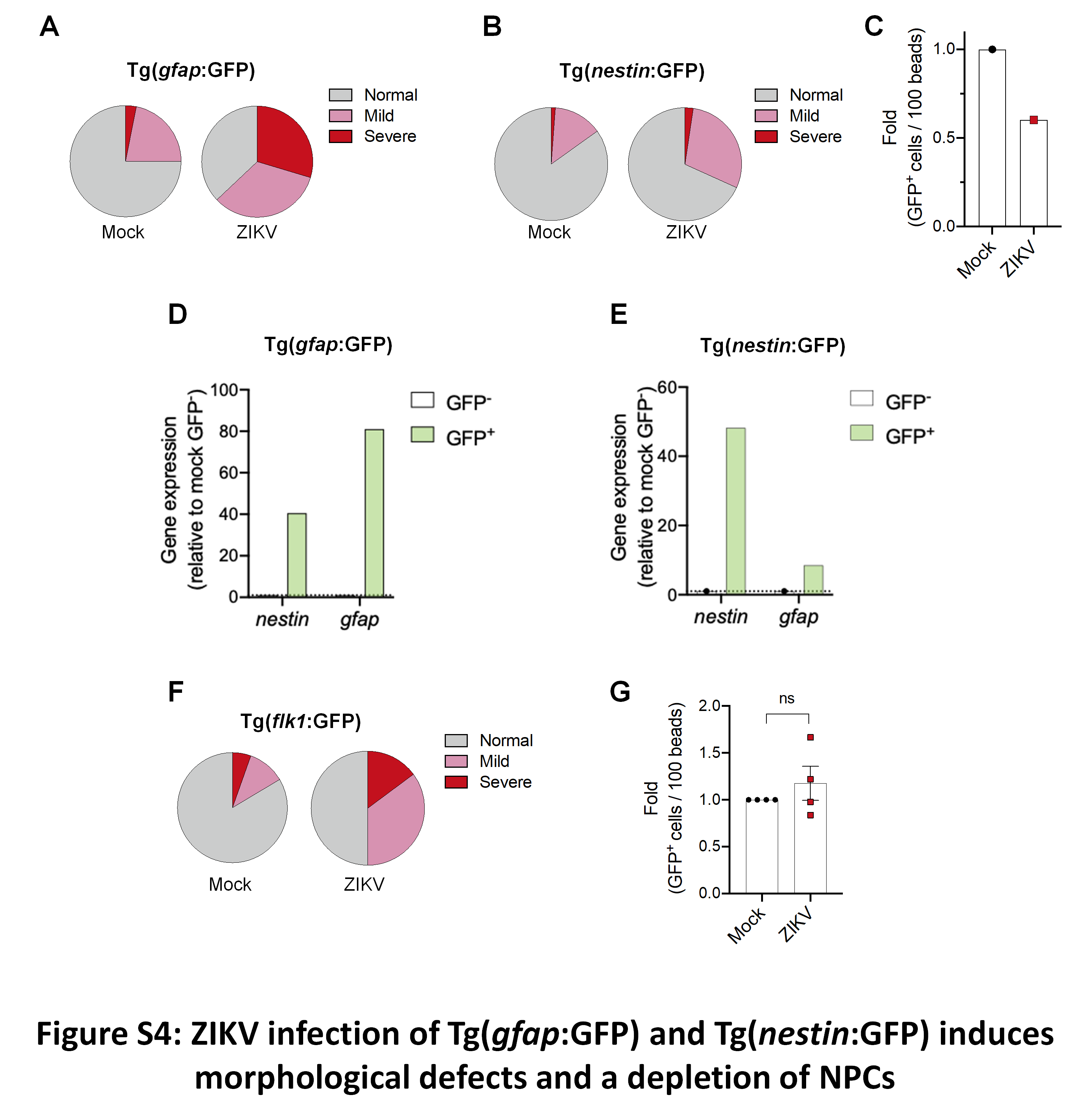

Supplement: S4 Fig — (A) Proportion of Tg(gfap:GFP) larvae with different morphological phenotypes at 3 days post-fertilization following ZIKV-injection (n = 27) or mock-injection (n = 32). N = 1. (B) Proportion of Tg(nestin:GFP) larvae with different morphological phenotypes at 3 days post-fertilization following ZIKV-injection (n = 85) or mock-injection (n = 87). N = 2. (C) At 1 day post-fertilization, ZIKV infected or uninfected whole transgenic Tg(nestin:GFP) embryos were dissociated in the presence of 10,000 fluorescent normalizing beads. Single cells and beads were counted by flow cytometry. The relative abundance of GFP+ cells counted per 100 beads are shown. (D-E) mRNA amounts of nestin and gfap in GFP+ (NPCs) and GFP- in mock and ZIKV-infected Tg(gfap:GFP) (D) and Tg(nestin:GFP) embryos (E) were analyzed at 1 dpf by RT-qPCR. (F) Within the two first hours following fertilization, Tg(flk1:EGFP) embryos were injected with ZIKV (n = 101) or vehicle (mock, n = 123). At 3 dpf, the morphology of the larvae was analyzed as in Fig 1. (G) At 1 day post-fertilization, ZIKV infected or uninfected whole transgenic Tg(flk1:EGFP) embryos were dissociated in the presence of 10,000 fluorescent normalizing beads. EGFP+ cells (i.e. endothelial cells) and beads were counted by flow cytometry. Data are shown as means ± SEM. (Mock, n = 95; ZIKV, n = 95; N = 4). ns: not significant; Student’s t-test. n indicates the number of fish; N represents the number of independent experimental repeats. (TIF) [file ppat.1012756.s004.tif]

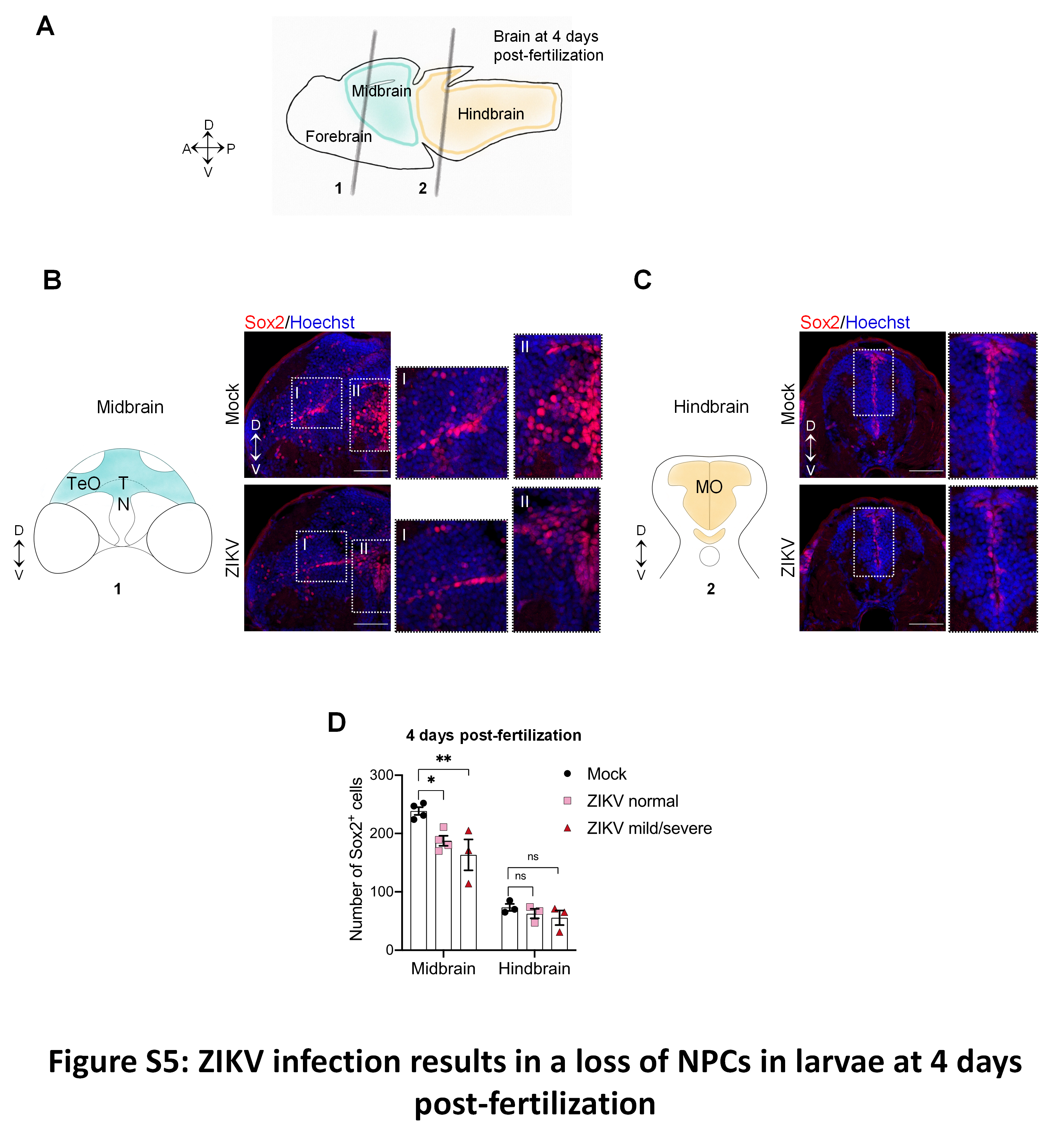

Supplement: S5 Fig — (A) Schematic representation of a zebrafish brain at 4 days post-fertilization. The three areas of the brain are shown: forebrain, midbrain and hindbrain. Gray lines represent the localization of the transverse sections. D = dorsal; V = ventral; A = anterior; P = posterior. (B-D) Number of neural progenitor cells (Sox2+ cells) in the midbrain (B) and the hindbrain (C) of 4 dpf mock-injected or ZIKV-injected fish. TeO = tectum opticum; T = midbrain tegmentum; N = region of the nucleus of medial longitudinal fascicle; MO = medulla oblongata. Scale bars = 50 μm. (D) Quantification of (B-C). Data are presented as means ± SEM. ** P ≤ 0.01; * P ≤ 0.05; ns: non-significant. Two-way ANOVA. (TIF) [file ppat.1012756.s005.tif]

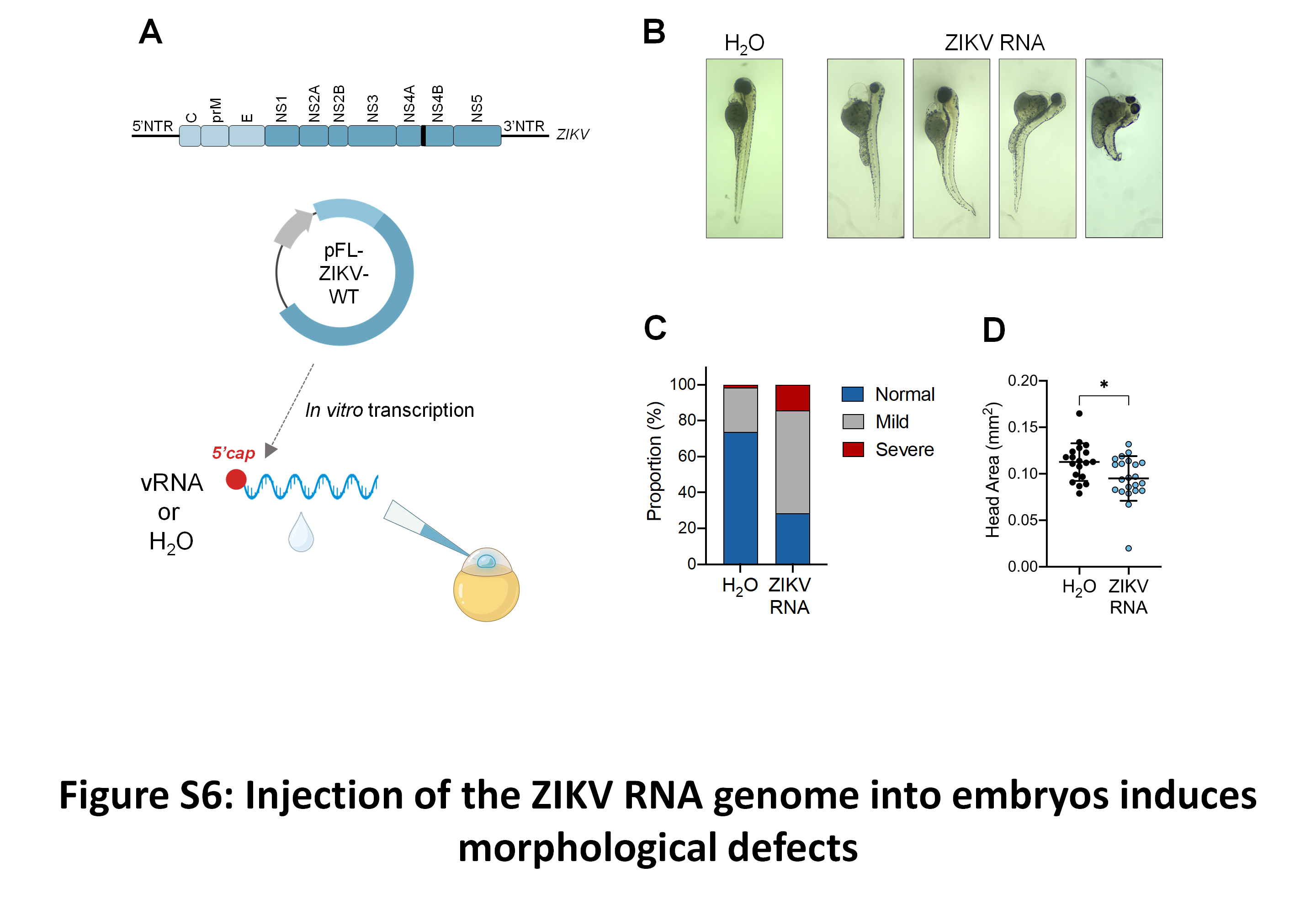

Supplement: S6 Fig — (A) In vitro transcribed ZIKV RNA genome (vRNA) was microinjected in the zebrafish embryo at 1 hour post-fertilization. Created with BioRender.com. (B) Representative pictures of microinjected larvae at 3 days post-fertilization. ZIKV vRNA injection induced both severe and mild developmental phenotypes. (C and D) Quantification of the proportion of larvae with the different phenotypes (C), and head size (D) at 3 dpf of the larvae (Mock, n = 19; ZIKV RNA, n = 22. N = 2). Data are means ± SEM. * P ≤ 0.05; Student’s t-test. n indicates the number of fish; N represents the number of experimental repeats. (TIF) [file ppat.1012756.s006.tif]
